# Marker-assisted breeding accelerates the development of multiple-stress-tolerant rice genotypes adapted to wider environments

**DOI:** 10.3389/fpls.2024.1402368

**Published:** 2024-07-12

**Authors:** Vignesh Mohanavel, Valarmathi Muthu, Rohit Kambale, Rakshana Palaniswamy, Prisca Seeli, Bharathi Ayyenar, Veeraranjani Rajagopalan, Sudha Manickam, Raghu Rajasekaran, Hifzur Rahman, Jagadeeshselvam Nallathambi, Manonmani Swaminathan, Gopalakrishnan Chellappan, Geethalakshmi Vellingiri, Raveendran Muthurajan

**Affiliations:** ^1^ Centre for Plant Molecular Biology and Biotechnology, Tamil Nadu Agricultural University, Coimbatore, Tamil Nadu, India; ^2^ Centre for Plant Breeding and Genetics, Tamil Nadu Agricultural University, Coimbatore, Tamil Nadu, India; ^3^ International Centre for Biosaline Agriculture, Dubai, United Arab Emirates; ^4^ Tamil Nadu Agricultural University, Coimbatore, Tamil Nadu, India

**Keywords:** rice, multiple-stress tolerance, abiotic and biotic, marker-assisted backcrossing, QTL pyramiding

## Abstract

**Introduction:**

Rice, one of the major staple food crops is frequently affected by various biotic/abiotic stresses including drought, salinity, submergence, heat, Bacterial leaf blight, Brown plant hopper, Gall midge, Stem borer, Leaf folder etc. Sustained increase of yield growth is highly necessary to meet the projected demand in rice production during the year 2050. Hence, development of high yielding and multiple stress tolerant rice varieties adapted to wider environments will serve the need.

**Methods:**

A systematic MAB approach was followed to pyramid eight major QTLs/genes controlling tolerance to major abiotic/biotic stresses viz., drought (*qDTY1.1* and *qDTY2.1*), salinity (*Saltol*), submergence (*Sub1*), bacterial leaf blight (*xa13* and *Xa21*), blast (*Pi9*) and gall midge (*Gm4*) in the genetic background of an elite rice culture CBMAS 14065 possessing high yield and desirable grain quality traits. Two advanced backcross derivatives of CBMAS 14065 possessing different combinations of target QTLs namely #27-1-39 (*qDTY1.1*+*qDTY2.1*+*Sub1*+*xa13*+*Xa21*+*Gm4+Pi9*) and #29-2-2 (*qDTY1.1*+*qDTY2.1*+*Saltol*+*Xa21*+*Gm4+Pi9*) were inter-mated.

**Results:**

Inter-mated F_1_ progenies harboring all the eight target QTLs/genes were identified through foreground selection. Genotyping of the inter-mated F_4_ population identified 14 progenies possessing all eight target QTLs/genes under homozygous conditions. All the fourteen progenies were forwarded up to F_8_ generation and evaluated for their yield and tolerance to dehydration, salinity, submergence, blast and bacterial leaf blight. All the 14 progenies exhibited enhanced tolerance to dehydration and salinity stresses by registering lesser reduction in their chlorophyll content, relative water content, root length, root biomass etc., against their recurrent parent Improved White Ponni/CBMAS 14065. All the 14 progenies harboring Sub1 loci from FR13A exhibited enhanced survival (90 - 95%) under 2 weeks of submergence /flooding when compared to their recurrent parent CBMAS 14065 which showed 100% susceptibility The inter-mated population showed a enhanced level of resistance to bacterial leaf blight (Score = 0 to 2) against blast (Score – 0) whereas the susceptible check CO 39 and the recurrent parent CBMAS 14065 recorded high level of susceptibility (Score = 7 to 9).

**Conclusion or discussion:**

Our study demonstrated the accelerated development of multiple stress tolerant rice genotypes through marker assisted pyramiding of target QTLs/genes using tightly linked markers. These multiple stress tolerant rice lines will serve as excellent genetic stocks for field testing/variety release and also as parental lines in future breeding programs for developing climate resilient super rice varieties.

## Introduction

Rice is a major cereal food crop to more than half of the global population (Khush and Jena, 2009; Roser et al., 2013), and rice production must be doubled by 2050 to meet the demand of the ever-growing global population (Yamori, 2013; Pandey et al., 2017). Current yield trends are insufficient to meet this demand, and declining land, water, labor, and nutrient resources pose further threats on global food security (Ray et al., 2013; Pradhan et al., 2019). Hence, increasing rice productivity is a key for achieving global food security.

Rice is cultivated in a wider range of environments and exposed to various abiotic and biotic stresses affecting yield (Pandey et al., 2017; Yugander et al., 2018). The changing climate aggravates the situation by increasing the occurrence of extreme weather events and changing the dynamics of pest and diseases. Abiotic stresses frequently affecting rice yields include drought, salinity, and submergence (Joneydi, 2012). Biotic stresses affecting rice yields include pests (brown plant hopper, leaf folder, stem borer, gall midge, etc.), pathogens (blast, bacterial leaf blight, false smut, etc.), and weeds. Crop losses due to major biotic stressors such as bacterial leaf blight, blast, and brown plant hopper are quite high (Hasan et al., 2015). In India, the area vulnerable to drought has increased by 57% since 1997, and the frequency of heavy rainfall and flash floods has increased by more than 80% since 2012 (The World Bank. 2023). Hence, a sustained increase in rice production depends on the development of resilient rice varieties tolerant to these biotic/abiotic stresses as well as requiring less labor, water, and nutrient resources.

Rice scientists persist in integrating crucial yield trait characteristics, including the 2Gs for the yield ceiling, by employing advanced breeding techniques, such as marker-assisted selection, haplotype-based breeding, and allele mining. The close proximity of the *GW7* gene for grain weight and the *DEP2* gene for grain number on chromosome 7 indicates the potential for simultaneous introgression of these two genes, provided that advantageous allelic variants exist in a single donor parent (Singh et al., 2024). Developing a multiple-stress-tolerant rice cultivar through conventional breeding approaches is practically very difficult. In conventional breeding, linkage drag continues to several generations and thus requires several backcross generations to eliminate undesirable traits. The discovery of major effect QTLs/genes controlling tolerance to major biotic/abiotic stresses, viz., drought, salinity, submergence, high temperature, bacterial leaf blight, blast, brown plant hopper, gall midge, low phosphorus, etc. (**Supplementary Table S1**) and advances in marker genotyping have paved the way for pyramiding of multiple QTLs/genes in a single genetic background through marker-assisted breeding (Janaki Ramayya et al., 2021).

In case of drought tolerance, *qDTY1.1* and *qDTY2.1* from a Philippine upland rice, Apo, have been used by several researchers to develop drought-tolerant versions of popular rice varieties (Venuprasad et al., 2009; Vikram et al., 2011; Muthu et al., 2020). Despite the existence of limited genetic variation for salinity tolerance traits in rice, intensive research efforts have identified a rice genotype, Pokkali, exhibiting a high level of tolerance to coastal salinity in India. Using Pokkali, a consistent QTL “*Saltol*” regulating Na/K uptake was identified and used by several researchers for the genetic improvement of salinity tolerance in rice (Singh et al., 2018). The discovery and utilization of a flood-tolerant rice “FR13A” discovered a mega effect of QTL “*Sub1*” regulating submergence tolerance in rice, which paved the way for deployment of MABB in rice (Neeraja et al., 2007).

Among the biotic stresses, blast is one of the most serious diseases causing yield reduction by up to 50% (Qu et al., 2006). Among >500 QTLs linked to blast resistance, only ~30 genes have been cloned and characterized (Das and Rao, 2015; Wu et al., 2016; Zheng et al., 2016). Out of this, *Pi9* and *Pi54* are the major genes offering broad-spectrum resistance against diverse isolates of *M. oryzae* (Qu et al., 2006). Against bacterial leaf blight (BLB) capable of causing 20%–40% yield reduction (Yasmin et al., 2017), 43 resistant genes have been identified (**Supplementary Table S1**). The combination of two or more resistant genes was found to be effective against prevailing strains of bacterial leaf blight in different locations of India. Pyramiding of *xa13+ Xa21*, either alone or in combination with other genes, viz., *Xa2*, *Xa5*, or *Xa4*, was proven effective in providing durable resistance to bacterial leaf blight. Gall midge is fast becoming a serious pest of rice in India, causing huge yield reduction. Several gall midge resistance genes have been identified (*Gm1*, *Gm2*, *gm3*, *Gm4*, *Gm5*, *Gm6*, *Gm7*, *Gm8*, *Gm9*, *Gm10*, and *Gm11*) and used in breeding rice against different biotypes of gall midge. Out of >10 Gm resistance genes, *Gm4* was reported to be effective against major biotypes of Gm (Divya et al., 2018).

Developing “climate-smart rice” involves the development of rice varieties that can endure increasingly severe and harsh circumstances in the future (Reay and Reay, 2019). It is anticipated that climate-smart rice will contribute to a sustainable rise in rice output under environmental constraints that regularly arise in a single growing season (Bin and Zhang, 2023). Molecular marker techniques are more effective for transferring desired gene(s) in any combination(s) into any desired genetic backgrounds. Hence, molecular techniques offer novel prospects for the enhancement of rice yields amid changing pest/disease/abiotic stress conditions. Molecular breeders have developed many high-yielding rice varieties with resistance against several abiotic and biotic stress tolerances (**Supplementary Table S2**). Multiple-stress-tolerant rice varieties can provide yield security in areas subjected to multiple stresses in a single season. However, there are only limited varieties with more than two tolerance/resistance traits (Dixit et al., 2020).

In this study, we attempted to pyramid eight different QTLs/genes conferring tolerance/resistance against drought (*qDTY1*.*1* and *qDTY2*.*1*), salinity (*Saltol*), submergence (*Sub1*), blast (*Pi9*), bacterial blight (*xa13* and *Xa21*), and gall midge (*Gm4*) in an elite genetic stock CBMAS 14065.

## Materials and methods

### Genetic materials used

A high-yielding, semi-dwarf, medium-duration, and medium slender grain rice culture CBMAS 14065 (derivative of a cross between the mega variety of South India, namely, Improved White Ponni and a drought-tolerant *indica* genotype Apo) was selected as the recurrent parent (Muthu et al., 2020). CBMAS 14065 harbors two major drought-tolerant QTLs (*qDTY1.1* and *qDTY2.1*) of Apo but exhibited susceptibility to salinity, submergence, bacterial leaf blight, and blast. Two intermittent genetic stocks (BC_2_F_1_s) of CBMAS 14065—namely: (1) CBMAS 14065 (27–1-39) harboring *qDTY1.1*, *qDTY2.1*, *Sub1*, *xa13*, *Xa21*, *Pi9*, and *Gm4* and (2) CBMAS 14065 (29–2-2) harboring *qDTY1*.*1*, *qDTY2*.*1*, *Saltol*, *Xa21*, *Pi9*, and *Gm4*—were used as parents for marker-assisted pyramiding strategy.

### Parental polymorphism survey and genotyping

A total of 75 markers located in the vicinity of the eight different target QTLs/genes, *viz*., *qDTY1.1*, *qDTY2.1*, *Saltol*, *Sub1*, *xa13*, *Xa21*, *Pi9*, and *Gm4*, were surveyed for genetic polymorphism between the respective donor parents and recurrent parent CBMAS 14065 (**Table 1**). Genomic DNA was extracted from leaf samples of all of the inter-mated progenies following the modified CTAB protocol (Tiwari et al., 2017). DNA quality was checked using agarose gel electrophoresis and quantified by using a Nanodrop ND-1000 spectrophotometer (Thermo Fisher Scientific, Wilmington, DE, USA). PCR reaction was carried out in a total volume of 10 μL containing 100 ng of DNA template, 6 μL of master mix (10x Taq Buffer, dNTPs, and Taq polymerase), and 0.5 μL each of 10 μM forward primer and reverse primers. All PCR reactions were amplified following the temperature profiles of initial denaturation at 94°C for 4 min, followed by 37 cycles of 94°C for 1 min, annealing at 55°C–60°C for 30 s, extension at 72°C for 1 min, and followed by a final extension at 72°C for 10 min using a thermal cycler (C1000 TOUCH PCR, Bio-Rad Inc., USA). PCR products were resolved in a 3% agarose gel stained with ethidium bromide, and the gel images were captured in a gel documentation system (Bio-Rad, USA).

**Table 1:** Details on the target traits, donors, QTLs/genes, linked markers, and chromosomal location(s).

Trait	Donors	QTLs/genes	Chromosome number	Position (Mb)	Foreground markers
Drought	Apo	*qDTY1.1*	1	34.9 - 37.8	RM472
		*qDTY2.1*	2	11.00–11.38	RM2634
Salinity	FL478	*Saltol*	1	10.6 -11.5	RM3412
Submergence	FR13A	*Sub1*	9	4.5 - 7.2	ART5
Blast	562–4 (CO43 pyramided with biotic stress)	*Pi9*	6	10.38	NBS4
Gall midge		*Gm4*	8	5.45	RM22550
Bacterial blight		*xa13*	8	26.7	xa13 Prom
		*Xa21*	11	20.5	pTA248

### Pyramiding QTLs/genes controlling tolerance to drought, salinity, submergence, bacterial leaf blight, blast, and gall midge

**Figure 1:**
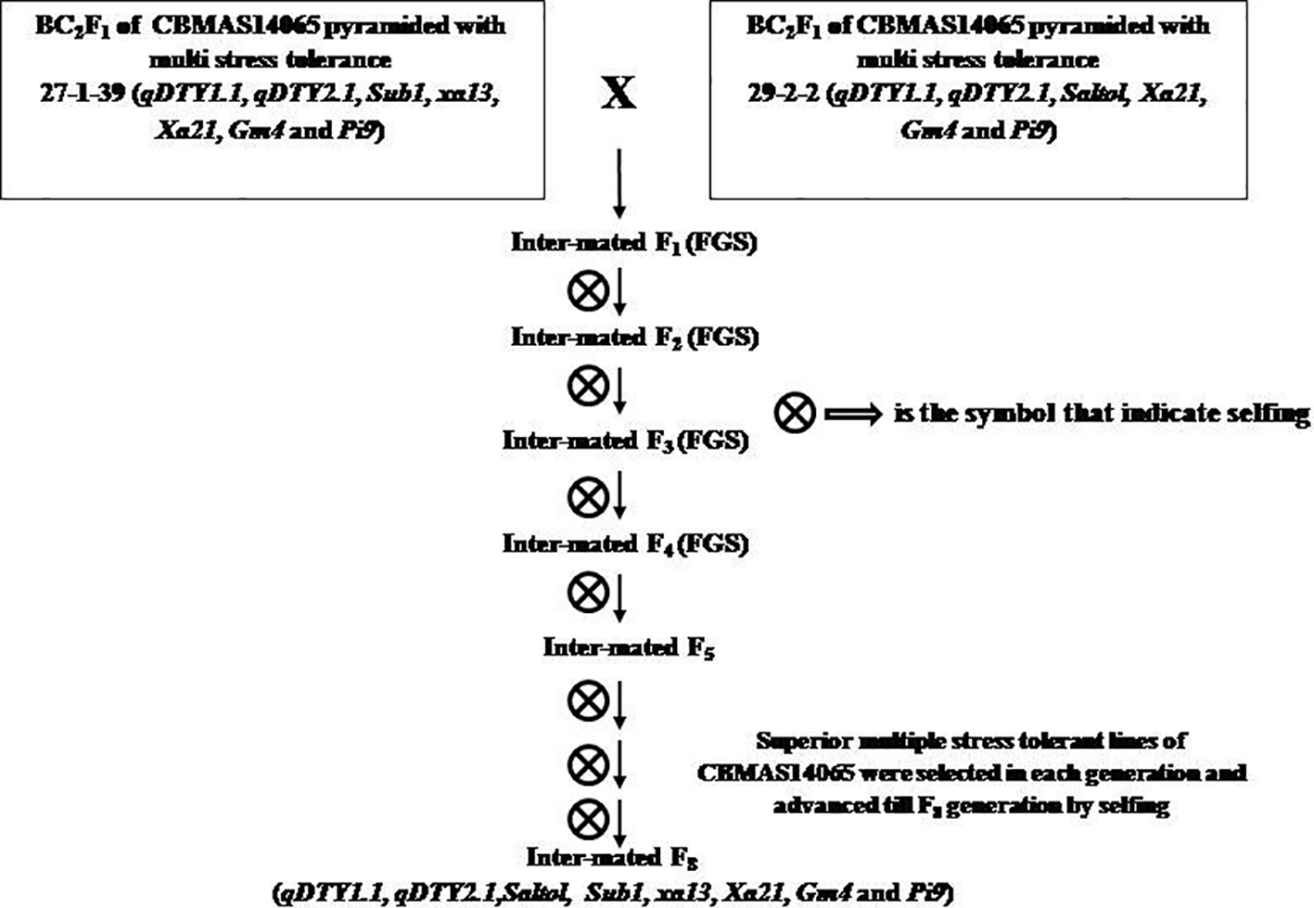
Breeding strategy followed for pyramiding 8 different QTLs/genes controlling tolerance against drought, salinity, submergence, blast, Bacterial leaf Blight and Gall midge in the genetic background of CBMAS14065.

Two intermittent genetic stocks were inter-mated, and F_1_ progenies harboring the entire eight target QTLs/genes were identified using foreground selection (**Figure 1**). True F_1_s were forwarded to further generations and positive progenies were identified through foreground selection.

### Phenotyping of inter-mated F_7_ progenies for the target traits

#### Evaluation for blast resistance

The 14 IMF_7_ lines were evaluated for their responses to blast pathogen along with the recurrent parent, donor parent (# 562–4), and susceptible check CO 39 under uniform blast nursery at Department of Rice, Tamil Nadu Agricultural University, Coimbatore, India. Each entry was raised on a nursery bed with a row length of 50 cm and row spacing of 10 cm. For uniform transmission of disease after inoculation, one row of the susceptible check (CO 39) was planted between every four entries and along the borders of the nursery bed. Young seedlings at the four-leaf stage were inoculated with fungal spores at a concentration of 1 × 10^5^ spores per milliliter using a fine nozzle sprayer, and high relative humidity was maintained in the nursery to favor disease development. The disease reaction in each progeny was recorded 35 days after sowing and periodically every 7 days until the susceptible check showed more than 80% of infection. Scoring of the disease reaction was done visually on a 0–9 SES scale according to the IRRI’s Standard Evaluation (SES) System (**Supplementary Table S3**).

#### Evaluation for bacterial leaf blight resistance

Inter-mated F_7_ progenies (IMF_7_ progenies) carrying bacterial blight resistance genes (*xa13* and *Xa21*) were grown in pots along with the donor and recurrent parents and subjected to artificial clip inoculation as described earlier (Kauffman, 1973). The bacterial leaf blight pathogen, *Xanthomonas oryzae pv. oryzae* (Xoo), was inoculated at a concentration of 10^9^ CFU/mL onto the rice plants at the maximum tillering stage (50–55 days). A total of 12–15 fully developed leaves from four plants in each progeny were selected and inoculated by artificial clipping procedure. The disease reaction was assessed at 21 days after inoculation as outlined in a previous study (Ramalingam et al., 2017). Lesion length was measured from the cut tip of the inoculated leaves and scored on a 0–9 SES scale (**Supplementary Table S4**). The scores of five infected leaves from each progeny were averaged to get the mean score.

#### Evaluation for salinity tolerance

A total of 14 progenies were raised under hydroponic conditions (Yoshida, 1976) along with the recurrent parent CBMAS 14065 and donor FL 478. A total of five plants were maintained for each entry. The pH of the Yoshida solution was monitored every day and maintained between 4 and 4.5. Then, 21-day-old seedlings were subjected to salinity stress by adding NaCl into the Yoshida solution at two different concentrations (75 and 100 mM), and a set of respective control plants was maintained without any stress. Visual symptoms of salt injury were measured at 21 days after salinization following the modified Standard Evaluation Scoring (SES, 1996) system (Sultana et al., 2022) (**Supplementary Table S5**). Phenotypic observations, such as leaf rolling, shoot length, root length, biomass, relative water content (RWC), and drying symptoms, were scored. The scores of five plants were averaged to get the mean score for each progeny.

#### Measuring responses against dehydration

A total of 14 inter-mated F_7_ progenies were raised under hydroponic conditions along with CBMAS 14065, Improved White Ponni, and Apo as described earlier. Dehydration stress was imposed on 21-day-old seedlings using PEG 6000 at two different concentrations, *viz*., 15% and 20% (Nahar et al., 2018), and a set of respective control plants was maintained without any stress. Visual symptoms of drought stress were measured at 21 days after the treatment following the modified Standard Evaluation Scoring (SES) system. Phenotypic observations such as leaf tip drying/rolling, shoot length, root length, relative water content (RWC), plant biomass, and dry symptoms were scored for both stressed and control plants. The scores from the five replications were averaged to get the mean score for each progeny.

#### Evaluation for submergence tolerance

Seedlings of IMF_7_ progenies, recurrent parent, and donor parent were raised in pots (three replications). Then, 21-day old seedlings were subjected to submergence stress by immersing the pots in a cement tank (the water level was maintained at a depth of 65 cm) containing water for 14 days. One pot was maintained under non-submerged conditions. Water depth was monitored every day to keep the plants under completely submerged conditions. After 14 days of submergence, the tank was de-submerged, and the plants were allowed to recover under natural conditions. At 14 days after de-submergence, the % plant survival (PPS), relative plant elongation under submergence (RPE), and chlorophyll reduction percentage of the plants were recorded using a SPAD meter (SPAD-502, Konica, Minolta, Germany). Data analysis and submergence scoring were assessed as per the earlier studies (Kaushal et al., 2018; Pradhan et al., 2019).

#### Evaluation of agronomic performance of IMF_7_ progenies

A total of 14 superior IMF_7_ progenies were evaluated for their agronomic performance along with the recurrent parent CBMAS 14065 and donor parents. A total of 13 plants per row and six rows per entry in 30 × 30-cm spacing were maintained. Different yield component traits, *viz*., plant height, days to first flowering, days to 50% flowering, number of tillers, flag leaf length and width, panicle length, 100-seeds weight, and single plant yield were recorded. All statistical analyses were carried out using Microsoft Excel 2021.

## Results

### Marker-assisted selection accelerated the pyramiding of QTLs/genes

Two BC_2_F_1_ progenies of CBMAS 14065—namely, #27–1-39 harboring seven different target QTLs/genes [namely, *qDTY1.1* and *qDTY2.1* (drought tolerance), *Sub1* (submergence tolerance), *xa13* and *Xa21* (bacterial leaf blight resistance), *Pi9* (blast resistance), and *Gm4* (gall midge resistance)] were crossed with another BC_2_F_1_ genetic stock of CBMAS 14065—namely #29–2-2 harboring six different QTLs/genes [namely *qDTY1.1* and *qDTY2.1* (drought tolerance), *Saltol* (salinity tolerance), *Xa21* (bacterial leaf blight resistance), *Pi9* (blast resistance), and *Gm4* (gall midge resistance)]. Foreground selection among 51 inter-mated F_1_s resulted in the identification of one plant #13 having all eight QTLs (*qDTY1.1* + *qDTY2.1* + *saltol* + *Sub1* + *xa13* + *Xa21* + *Pi9* + *Gm4)* (**Table 2**) which was selfed and forwarded further. The foreground selection of 43 F_2_ progenies identified one progeny—namely, #13-31—harboring five loci under homozygous conditions and three loci under heterozygous conditions and three other progenies harboring four loci under homozygous conditions and with remaining four loci under heterozygous conditions (**Table 2**).

**Table 2:** Foreground selection of inter-mated progenies of CBMAS 14065.

Plant no.	*qDTY1.1*	*qDTY2.1*	*Saltol*	*Sub1*	*xa13*	*Xa21*	*Pi9*	*Gm4*	QTLs/genes
Inter-mated F_1_									
13	+	+	H	H	H	H	+	H	8
F_2_ generation									
Genotypes									
13–10	+	+	H	H	H	+	+	H	8
13–11	+	+	H	H	+	H	+	H	8
13–31	+	+	H	+	H	+	+	H	8
13–33	+	+	H	H	H	H	+	+	8
F_3_ generation									
Genotypes									
13–31-11	+	+	+	+	H	+	+	+	8
13–31-24	+	+	+	+	+	+	+	H	8
13–31-26	+	+	H	+	+	+	+	+	8
13–31-56	+	+	+	+	+	+	+	H	8
13–31-122	+	+	+	+	+	+	+	H	8

+, homozygous donor allele; -, homozygous susceptible allele; H, heterozygotes.

IMF_2_ progeny #13–31 harboring five loci under homozygous conditions was forwarded to the next generation. The foreground genotyping of 134 IMF_3_ progenies identified a total of five IMF_3_ progenies, *viz*., 13–31-11, 13–31-24, 13–31-26, 13–31-56, and 13–31-122, harboring seven loci under homozygous conditions and one loci under heterozygous condition in different combinations (**Table 2**).

#### Evaluation of inter-mated F_4_

**Figure 2:**
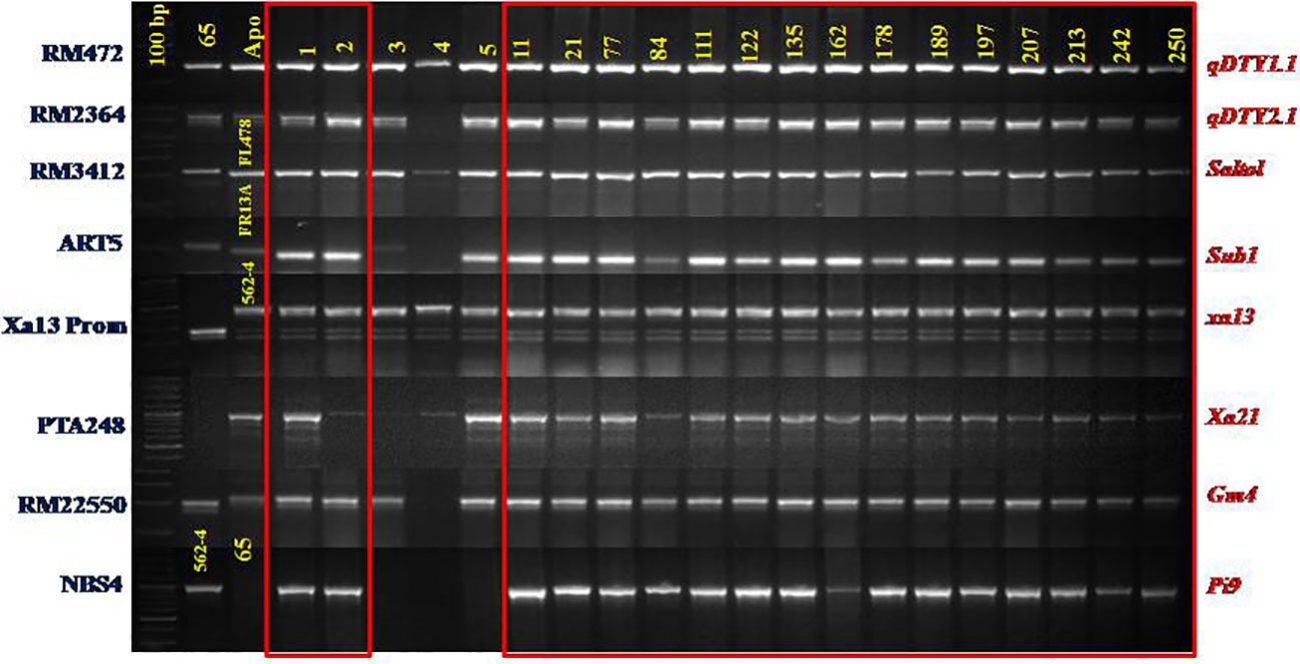
Foreground selection of IMF_4_ progenies of CBMAS 14065 using markers liked to 8 target QTLs/Genes.

An evaluation of 600 IMF_4_ progenies identified a total of 14 progenies harboring all of the eight target loci under homozygous conditions (**Table 3**; **Figure 2**). Upon evaluation of 14 IMF_5_, a total of 70 superior plants (five progenies from each of the 14 families) were selected based on their agronomic traits and forwarded to IMF_6_–IMF_7_ generations.

**Table 3:** Foreground selection of 14 IMF_4_ progenies of CBMAS 14065 showing the homozygosity of eight different target loci.

P. no.	*qDTY1.1*	*qDTY2.1*	*Saltol*	*Sub1*	*xa13*	*Xa21*	*Pi9*	*Gm4*	QTLs/genes
11	+	+	+	+	+	+	+	+	8
21	+	+	+	+	+	+	+	+	8
77	+	+	+	+	+	+	+	+	8
84	+	+	+	+	+	+	+	+	8
111	+	+	+	+	+	+	+	+	8
122	+	+	+	+	+	+	+	+	8
135	+	+	+	+	+	+	+	+	8
162	+	+	+	+	+	+	+	+	8
178	+	+	+	+	+	+	+	+	8
189	+	+	+	+	+	+	+	+	8
197	+	+	+	+	+	+	+	+	8
207	+	+	+	+	+	+	+	+	8
213	+	+	+	+	+	+	+	+	8
242	+	+	+	+	+	+	+	+	8
250	+	+	+	+	+	+	+	+	8

### Phenotyping of IMF_7_ progenies of CBMAS 14065 for the target traits

#### Responses to dehydration

**Figure 3:**
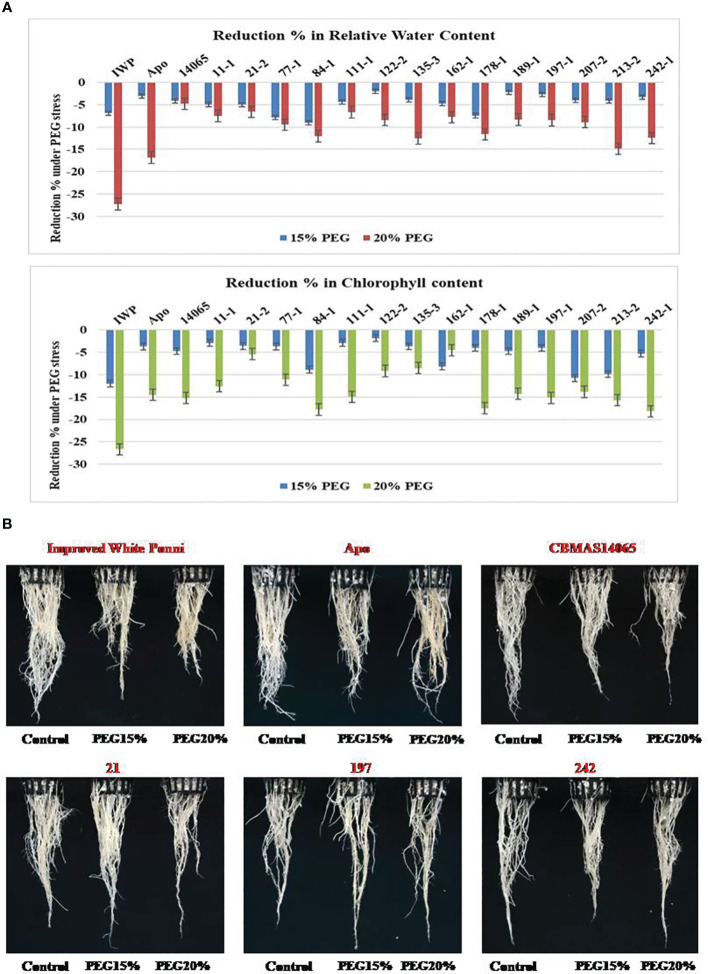
**(A)** Graphical representation of intermated F_7_ progenies of CBMAS14065 against drought tolerance using PEG. **(B)** Root growth behaviour of intermated F_7_ progenies of CBMAS14065 under dehydration induced by PEG6000 treatment.

At 21 days after imposing dehydration stress, CBMAS 14065 and all of the 14 inter-mated F_7_ progenies harboring *qDTY1.1* and *qDTY2.1* exhibited enhanced tolerance compared to the susceptible check Improved White Ponni. Four progenies (21–2, 122–2, 197–1, and 242–1) exhibited a lesser reduction of root (length and biomass) and shoot (length and biomass) growth-related traits under dehydration (**Table 4**; **Figures 3A, B**). The susceptible parent Improved White Ponni showed >25% reduction in its chlorophyll content and relative water content, whereas the tolerant parent Apo, recurrent parent CBMAS 14065, and IMF_7_ progenies showed a maximum of 15% reduction in their chlorophyll content and relative water content (**Figure 3A**).

**Table 4:** Evaluation of inter-mated F_7_ progenies of CBMAS 14065 for dehydration tolerance using PEG.

Genotypes	Shoot length (% reduction)		Root length (% reduction)		Root fresh biomass (% reduction)		Root dry biomass (% reduction)		Shoot fresh biomass (% reduction)		Shoot dry biomass (% reduction)	
	PEG conc.		PEG conc.		PEG conc.		PEG conc.		PEG conc.		PEG conc.	
	15%	20%	15%	20%	15%	20%	15%	20%	15%	20%	15%	20%
**IWP**	7.08	11.48	17.45	37.74	22.35	34.54	21.12	30.35	12.04	32.07	14.03	45.70
**Apo**	5.94	7.54	7.18	10.26	5.20	7.25	8.78	7.80	4.14	10.21	4.50	17.10
**CBMAS 14065**	4.61	6.83	7.35	9.80	6.02	8.36	7.17	15.00	4.08	7.82	4.41	18.19
**11–3**	3.55	4.80	2.65	3.54	3.21	9.38	5.37	7.80	5.86	16.35	10.67	12.20
**21–2**	3.50	4.55	5.56	10.26	3.49	9.97	8.10	10.00	6.14	8.24	4.72	15.68
**77–1**	3.17	7.13	7.94	27.78	2.66	14.98	7.36	19.91	6.23	18.52	5.57	24.36
**84–1**	3.36	4.20	8.26	9.50	2.06	13.17	12.41	25.82	4.85	13.87	4.19	21.01
**111–1**	3.52	5.99	7.84	13.33	5.92	6.98	8.24	11.76	6.88	12.27	6.01	11.85
**122–2**	3.40	8.84	4.03	9.68	3.50	9.79	5.24	11.19	5.88	10.08	5.64	19.42
**135–3**	3.21	7.54	4.48	7.62	3.92	12.23	9.09	10.45	8.31	18.97	8.47	16.46
**162–1**	3.82	4.40	8.85	11.06	6.44	16.24	9.76	14.63	8.79	12.15	8.78	14.68
**178–1**	3.31	6.28	7.60	13.31	4.51	13.44	6.25	20.14	7.87	16.77	5.79	13.47
**189–1**	3.01	11.75	7.91	16.21	5.08	14.24	6.42	16.98	7.51	10.29	5.12	11.35
**197–1**	3.37	4.89	7.69	9.50	4.88	9.32	8.00	26.00	8.42	10.56	7.28	19.44
**207–2**	3.96	4.36	8.81	11.89	4.30	15.74	6.45	9.14	9.16	16.69	7.88	11.49
**213–2**	4.21	6.95	8.40	13.03	5.81	17.54	7.60	23.98	9.28	19.94	9.46	16.22
**242–1**	2.95	8.70	6.31	9.46	5.59	9.57	8.90	14.38	5.35	10.97	6.94	15.17

SL, shoot length; RL, root length; FRB, fresh root biomass; DRB, dry root biomass; SB, fresh shoot biomass; DSB, dry shoot biomass.

#### Phenotypic evaluation for salt tolerance

**Figure 4:**
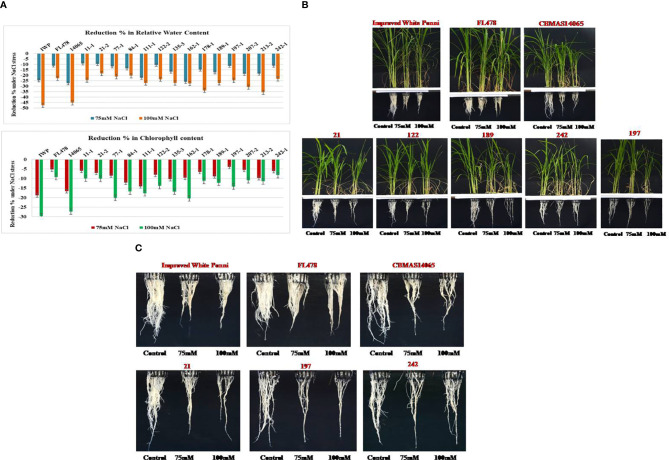
**(A)** Percent reduction in chlorophyll content and relative water content in the leaves of intermated F_7_ progenies of CBMAS14065 against NaCI stress. **(B)** Responses of rice genotypes against salinity stress induced by NaCI (Control-without stress; 75mM and 100mM refers to NaC1 contrentration). **(C)** Salinity responsive changes in the root growth pattern of inter-mated progenies of CBMAS14065.

Inter-mated F_7_ progenies of CBMAS 14065 harboring eight target QTLs, including the salinity tolerance QTL, namely, “*Saltol*”, were grown under hydroponic conditions and subjected to NaCl stress (75 and 100 mM). After 21 days of salinity, scoring was done based on the symptoms of salt injury using a modified standard evaluation system (SES) developed at IRRI, Philippines. The susceptible check IWP and the recurrent parent CBMAS 14065 exhibited more than 40% reduction in their relative water content and >50% reduction in the shoot–root dry biomass (**Table 5**; **Figures 4A–C**). Four progenies, *viz*., #21–2, #122–2, #197–1, and #242–1), exhibited a high level of tolerance to salinity by recording lesser % reduction in terms of chlorophyll content and relative water content.

**Table 5:** Salinity-responsive changes in the shoot/root growth pattern among the inter-mated F_7_ progenies CBMAS 14065.

Progenies	Shoot length (% reduction)		Root length (% reduction)		Root fresh biomass (% reduction)		Root dry biomass (% reduction)		Shoot fresh biomass (% reduction)		Shoot dry biomass (% reduction)	
	NaCl		NaCl		NaCl		NaCl		NaCl		NaCl	
	75 mM	100 mM	75 mM	100 mM	75 Mm	100 mM	75 mM	100 mM	75 mM	100 mM	75 mM	100 mM
IWP	26.67	32.08	32.29	42.71	55.19	59.07	56.03	65.44	56.03	65.44	52.65	63.21
CBMAS 14065	17.66	22.79	24.00	41.33	48.78	57.47	38.25	42.98	38.25	42.98	39.51	48.84
FL478	7.94	16.77	9.27	13.91	18.72	31.35	19.76	40.03	19.76	40.03	17.13	37.77
11–3	7.89	19.17	7.60	18.80	28.18	53.04	16.15	55.08	16.15	55.08	19.36	85.00
21–2	7.77	12.23	5.17	6.90	10.27	37.50	13.21	34.46	13.21	34.46	26.72	36.21
77–1	8.43	12.84	8.33	14.17	21.18	53.20	27.80	54.63	27.80	54.63	29.75	56.67
84–1	8.38	14.23	8.30	15.77	13.33	29.44	19.92	33.73	19.92	33.73	17.07	43.90
111–1	14.17	15.37	8.71	14.94	44.94	51.97	57.20	58.70	57.20	58.70	27.67	27.89
122–2	7.78	17.76	7.20	13.98	10.63	37.50	14.88	36.61	14.88	36.61	16.38	42.33
135–3	13.57	15.80	11.76	16.81	20.00	40.00	16.78	49.43	16.78	49.43	25.27	61.76
162–1	14.18	23.09	8.97	22.42	16.54	42.31	16.69	46.57	16.69	46.57	7.09	67.40
178–1	6.38	11.52	10.50	9.66	19.25	35.83	13.20	30.23	13.20	30.23	4.35	28.26
189–1	11.37	14.51	9.56	18.33	11.83	53.25	19.69	48.31	19.69	48.31	36.46	68.13
197–1	7.88	16.77	6.22	13.33	10.85	30.23	15.64	40.18	15.64	40.18	18.75	44.79
207–2	14.59	18.43	9.50	11.16	28.35	35.05	33.92	22.99	33.92	22.99	30.90	30.62
213–2	10.83	20.83	12.56	26.57	18.52	47.41	25.74	61.14	25.74	61.14	60.34	62.38
242–1	7.55	13.27	6.98	11.63	9.66	31.03	12.78	30.22	12.78	30.22	14.03	43.11

SL, shoot length; RL, root length; FRB, fresh root biomass; DRB, dry root biomass; FSB, fresh shoot biomass; DSB, dry shoot biomass.

#### *Sub1* introgressed lines exhibited enhanced tolerance against submergence

**Figure 5:**
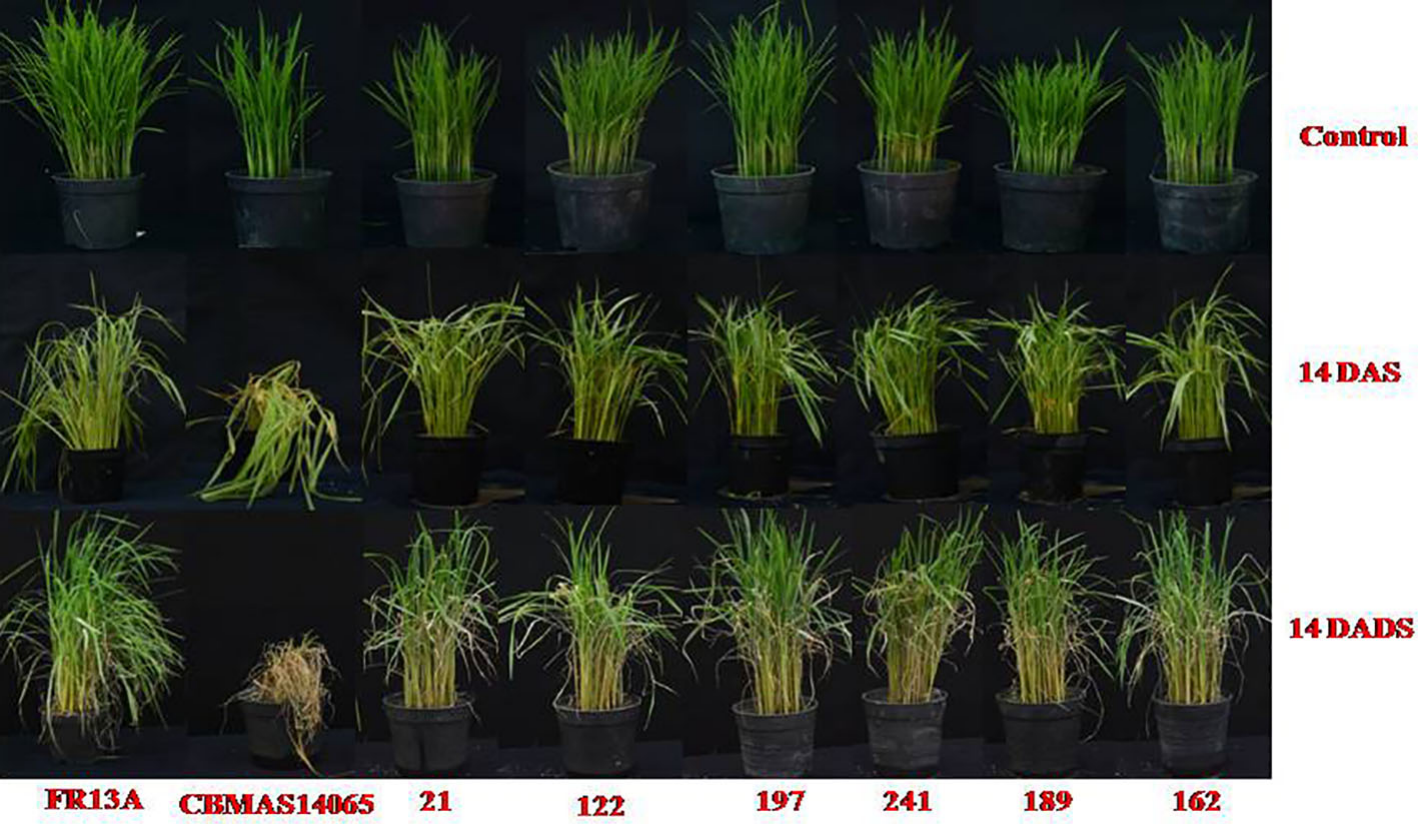
Responses of inter-mated F_7_ progenies against 14 days of submergence stress. Control, no submerge; DAS refers to Days After Submergence; DADS refers to Days after De-Submergence.

The 21-day-old seedlings of 14 inter-mated F_7_ progenies of CBMAS 14065 grown in pots were exposed to submergence stress for 14 days in cement tanks along with their parents and appropriate checks and then de-submerged. After a stress period of another 21 days, parameters like plant height, number of plants, number of leaves, and chlorophyll content were measured before and after submergence to calculate the regeneration capacity of the progenies. Submergence-stress-tolerant progenies were identified based on their recovery/regeneration percentage. After 14 days of de-submergence, the recurrent parent CBMAS 14065 showed 100% mortality, whereas all of the 14 inter-mated F_7_ progenies showed enhanced tolerance (90%–100%) against 14 days of submergence, which was on par with the donor parent FR13A (**Table 6**; **Figure 5**). Relative plant elongation (RPE) during submergence was high in the recurrent parent CBMAS 14065 (123.42%), whereas the progenies exhibited significantly lesser elongation (80.69% to 113.64%) equal to the donor parent FR13A (**Table 6**).

**Table 6:** Responses of inter-mated F_7_ progenies against submergence.

Progenies	Relative plant elongation (RPE) (%)	Reduction % chlorophyll	Percentage plant survival (PPS) (%)
CBMAS 14065	123.42	100.00	0
FR13A	110.84	9.13	100
11–3	96.39	9.96	92
21–2	111.72	7.46	96
77–1	93.15	5.88	92
84–1	111.45	5.98	92
111–1	97.86	9.04	90
122–2	94.67	5.54	96
135–3	106.38	10.07	94
162–1	102.90	10.24	92
178–1	98.57	10.20	92
189–1	80.69	10.17	90
197–1	106.06	8.14	94
207–2	113.64	9.70	90
213–2	107.36	10.00	92
241–2	89.49	9.60	96

#### Introgression of *Pi9* conferred enhanced resistance to blast

**Figure 6:**
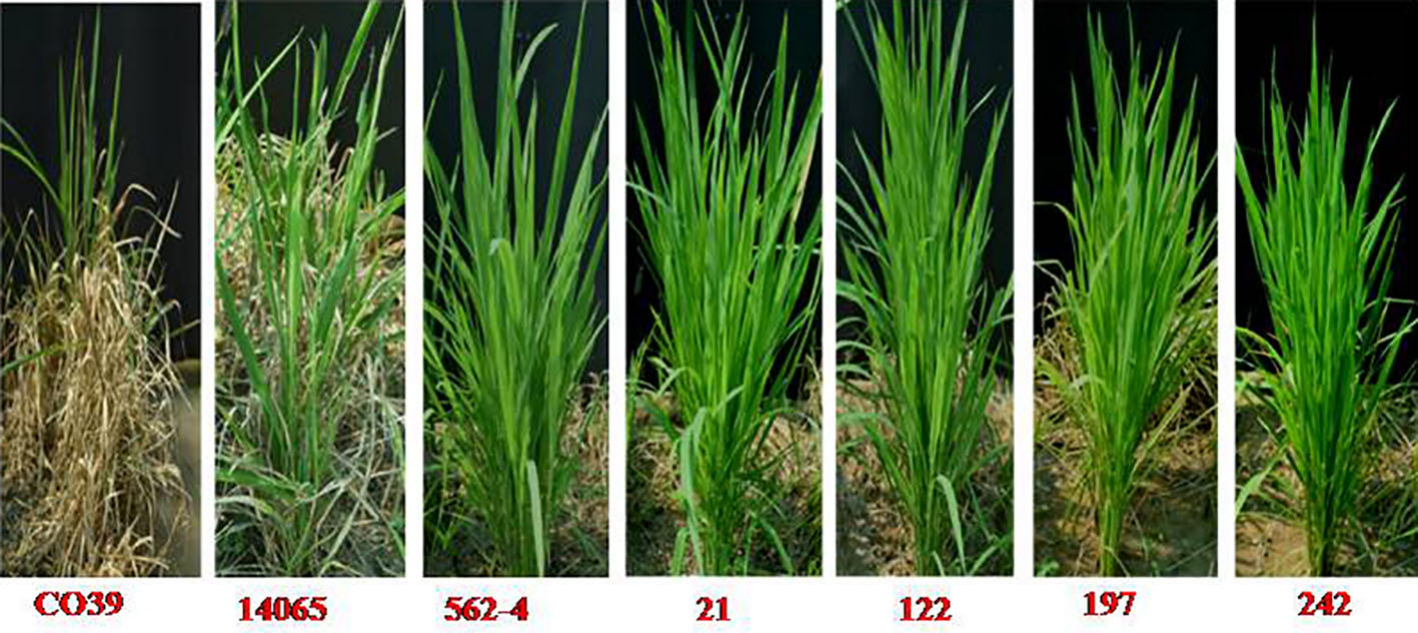
Responses of recurrent parent (CBMAS14065), donor (#562-4) and inter-mated F_7_ progenies against blast pathogen.

All of the 14 inter-mated F_7_ progenies harboring a broad-spectrum blast resistance gene, *Pi9*, were screened under uniform blast nursery along with the recurrent parent (CBMAS 14065), donor parent (562–4), and susceptible check (CO 39) during the winter of 2021. All of the 14 inter-mated F_7_ progenies of CBMAS 14065 exhibited a high level of resistance to blast with a score of “0” comparable to the donor, #562–4. Recurrent parent CBMAS 14065 exhibited a high level of susceptibility with a score of 5 to 7 (**Table 7**; **Figure 6**).

**Table 7:** Evaluation of inter-mated F_7_ progenies of CBMAS 14065 against blast.

Generation	Sample no.	SES scoring (35 days after inoculation)	SES scoring (50 days after inoculation)	Tolerance level
Parents	CBMAS 14065	7	8	Susceptible
	562–4	0	0	Resistant (R)
IMF_7_	11–3	0	0	Resistant (R)
	21–2	0	0	Resistant (R)
	77–1	0	0	Resistant (R)
	84–1	0	0	Resistant (R)
	111–1	0	0	Resistant (R)
	122–2	0	0	Resistant (R)
	135–3	0	0	Resistant (R)
	162–1	0	0	Resistant (R)
	178–1	0	0	Resistant (R)
	189–1	0	0	Resistant (R)
	197–1	0	0	Resistant (R)
	207–2	0	0	Resistant (R)
	213–2	0	0	Resistant (R)
	241–1	0	0	Resistant (R)

#### Responses of inter-mated F_7_ progenies against bacterial leaf blight

**Figure 7:**
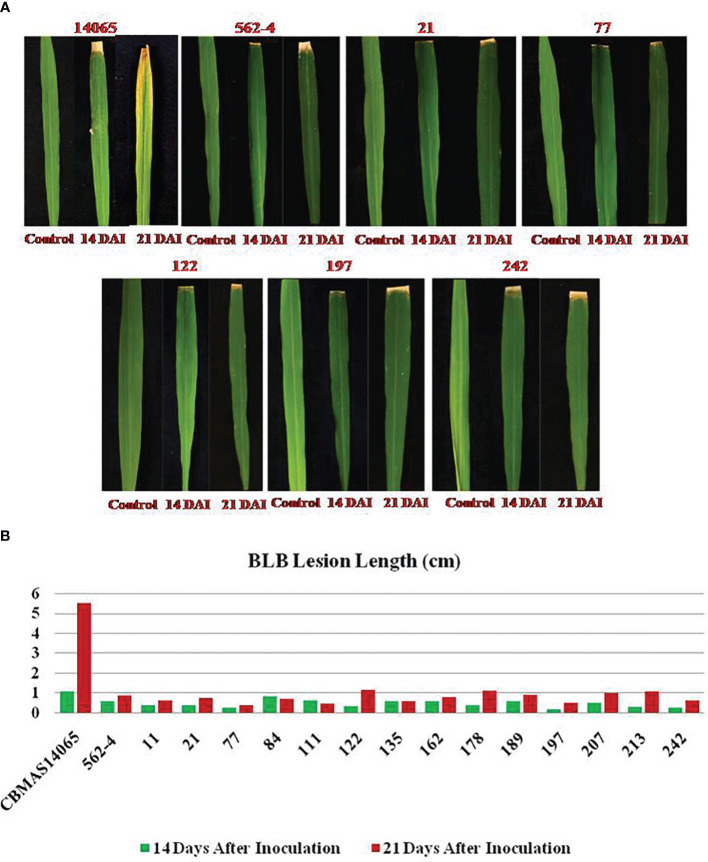
**(A)** Response of inter-mated of CBMAS14065 harboring *xx13* and *Xa21* and the parents against BLB infection. **(B)** Responses of inter-mated F_7_ progenies of CBMAS14065 harboring *xx13* and *Xa21* and the parents against BLB infection; Graphical representation of lesion of lesion at 14DAI and 21 DALI.

The inter-mated F_7_ progenies of CBMAS 14065 pyramided with eight different target loci, including two bacterial leaf blight resistance loci—namely, *xa13* and *Xa21*—exhibited a high level of resistance (lesion length of 0.3–1.0 cm) similar to that of the donor parent #562–4, whereas the recurrent parent showed high susceptibility with a leaf lesion length of 1.1–5.5 cm (**Figures 7A, B**).

#### Agronomic performance of inter-mated F_7_ progenies

**Figure 8:**
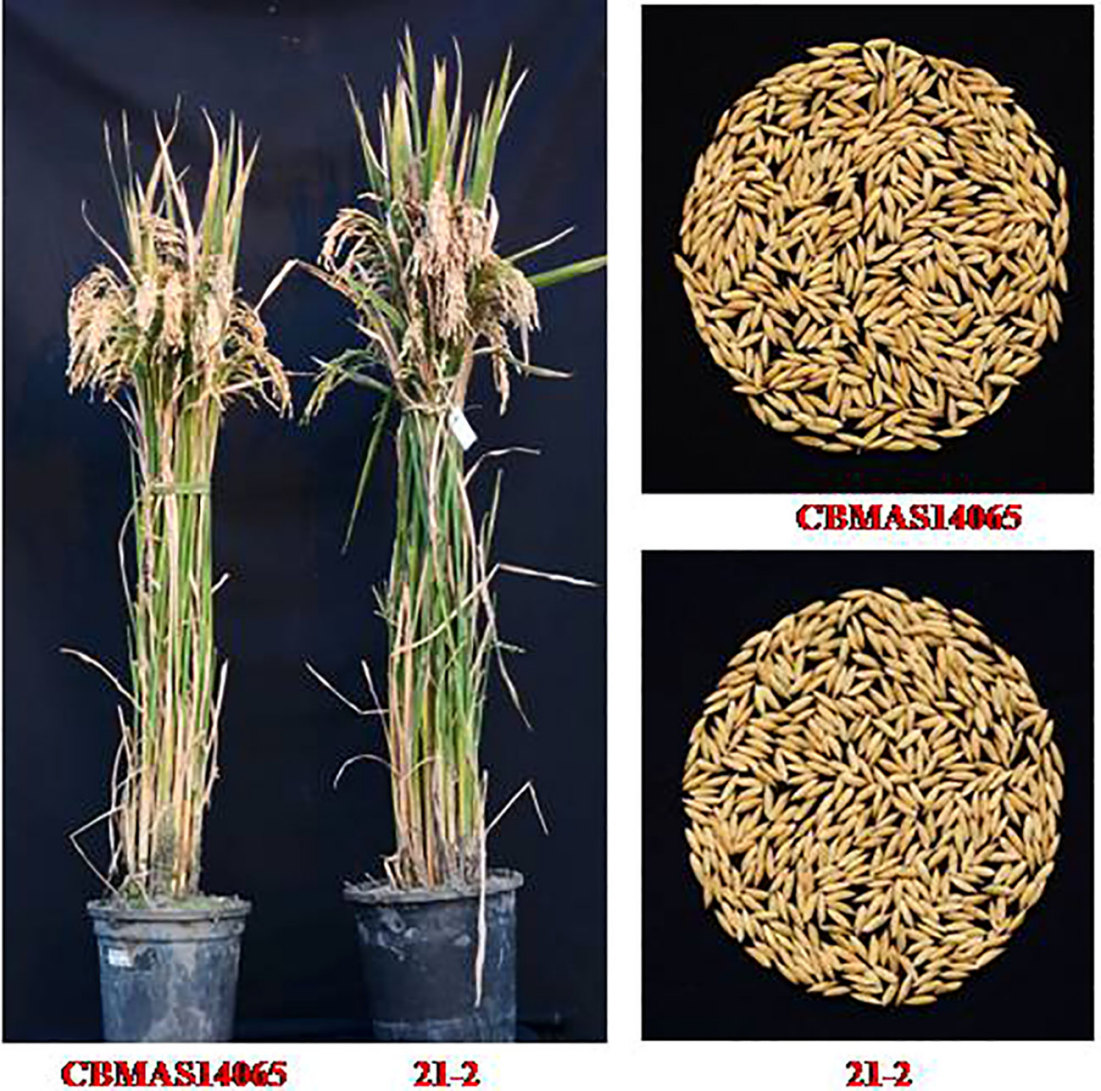
Photograph showing the planet morphology and grain characteristics of an IMF_7_ progeny along with recurrent Parent CBMAS14065.

A total of 14 inter-mated progenies pyramided with eight different target loci were evaluated for their agronomic performance over two seasons (Kharif 2020 and Kharif 2021) along with the recurrent parent CBMAS 14065, Improved White Ponni, and relevant donors. Days to flowering among the 14 inter-mated progenies ranged between 96 to 103 days, while the recurrent parent CBMAS 14065 flowered in 113 days. The inter-mated progenies were found to possess a higher number of productive tillers (32 to 48 tillers) when compared to CBMAS 14065 (30 tillers). Overall, the inter-mated progenies of CBMAS 14065 were found to possess a higher yield potential (60 to 116 g/plant) when compared to CBMAS 14065 (58 g/plant). The 100-grain weight of inter-mated progenies ranged between 1.124 to 1.570 g when compared to 1.249 g recorded by CBMAS 14065. Overall, the pyramided IMF_7_ lines were found to be 10 days earlier than CBMAS 14065 in duration and 22–42 cm more dwarf than the popular variety Improved White Ponni (**Table 8**; **Figure 8**).

**Table 8:** Agronomic performance of the 14 inter-mated F7 progenies of CBMAS14065 pyramided with multiple-stress-tolerant QTLs/genes.

Plant no.	Days to flowering (first)	Plant height (cm)	Number of tillers	Panicle length (cm)	100-grains weight (gm)	Grain yield/plant (gm)
CBMAS14065	113	100	28	21	1.249	58
Improved White Ponni	119	130	24	24.5	1.725	47
11–3	98^a^	100	30^a^	19.5	1.411^a^	72^a^
21–2	99^a^	108	23	20.5	1.245	90^a^
77–1	99^a^	102	42^a^	21.0	1.338^a^	77^a^
84–1	98^a^	96^a^	36^a^	21.5	1.125	70^a^
111–1	100^a^	99	45^a^	20.4	1.523^a^	87^a^
122–2	96^a^	98	37^a^	20.0	1.514^a^	112^a^
135–3	98^a^	88^a^	36^a^	20.0	1.433^a^	85^a^
162–1	96^a^	92^a^	33^a^	20.0	1.570^a^	78^a^
178–1	102^a^	88^a^	37^a^	22.0^a^	1.432^a^	87^a^
189–1	103^a^	91^a^	40^a^	22.5^a^	1.439^a^	83^a^
197–1	99^a^	105	33^a^	22.5^a^	1.429^a^	94^a^
207–2	102^a^	100	26	21.0	1.393^a^	80^a^
213–2	101^a^	99	45^a^	22.5^a^	1.124	85^a^
241–1	100^a^	100	48^a^	22.0^a^	1.456^a^	116^a^
SE	0.570	1.609	2.120	0.284	0.036	3.579
CD	1.232	3.475	4.579	0.614	0.079	7.732

a Significance at 5% level (than CBMAS14065).

## Discussion

Most of the rice varieties with high yields are susceptible to both biotic and abiotic stressors. Conventional breeding methods have been utilized effectively to produce numerous crop varieties with improved yield, but conventional breeding faces challenges (time-consuming and labor-intensive process) in pyramiding multiple-component traits/genomic loci conferring tolerance to various biotic/abiotic stresses. Recent advances in genotyping platforms and marker-assisted breeding have provided opportunities to pyramid or mobilize multiple QTLs/genes from several donors simultaneously into a single genetic background (Ramalingam et al., 2017; Das et al., 2018; Kumar et al., 2018; Rana et al., 2019; Valarmathi et al., 2019; Dixit et al., 2020; Muthu et al., 2020; Pancaldi and Trindade, 2020). Similar approaches have been successfully demonstrated in mobilizing QTLs/genes for various biotic (blast, bacterial leaf blight, and gall midge) and abiotic (salinity and submergence) stresses in the genetic background of popular rice varieties, *viz*., Naveen, Lalat, and Tapaswini (Das and Rao, 2015; Kumar et al., 2018; Janaki Ramayya et al., 2021).

CBMAS 14065 was developed as a semi-dwarf version of Improved White Ponni, and during field testing, this culture was found to exhibit a high level of susceptibility to salinity, submergence, blast, and bacterial leaf blight. Hence, attempts were made to pyramid tolerance to salinity (*Saltol*), submergence (*Sub1*), bacterial leaf blight (*xa13* and *Xa21*), blast (*Pi9*), and gall midge (*Gm4*) along with the drought tolerance loci *qDTY1.1* and *qDTY2.1* already present in it. Except for drought and salinity tolerance, gene-based markers were used to monitor the introgression of genetic loci controlling tolerance to submergence, bacterial blight, and blast. Adequate numbers of progenies (~800 to 1,000) were maintained in each generation for the effective selection of targeted gene combinations. In the IMF_4_ generation, elite lines harboring all of the target genes/QTLs under homozygous conditions were identified and forwarded further through selfing. Under each generation, the presence of target loci was confirmed through foreground selection using trait-linked markers and yield potential.

Drought stress is a major problem affecting rice cultivation under both irrigated and rain-fed conditions, causing serious yield losses to the farmers. The discovery of major-effect QTLs, *viz*., *qDTY1.1*, *qDTY2.1*, *qDTY12.1*, etc., in Apo, Nagina 22, Adaysel, Way rarem, etc., enabled the deployment of marker-assisted breeding to improve drought tolerance or yield under drought in rice (Vikram et al., 2011; Ghimire et al., 2012; Mishra et al., 2013). In the present study, CBMAS 14065 and the inter-mated F_7_ population of CBMAS 14065 harboring *qDTY1.1* and *qDTY2.1* were screened for their tolerance to dehydration using polyethylene glycol (6000). CBMAS 14065 and the inter-mated F_7_ progenies pyramided with *qDTY1.1* and *qDTY2.1* recorded lesser reduction in their shoot length, root length, relative water content, root fresh biomass, root dry biomass, shoot fresh biomass, and shoot dry biomass during dehydration as compared to Improved White Ponni. This study has demonstrated the importance of drought-tolerant QTLs (*qDTY1.1* and *qDTY2.1*) in alleviating drought-induced dehydration and other negative impacts similar to earlier reports (Venuprasad et al., 2009; Ghimire et al., 2012; Vikram et al., 2015).

Rice exhibits high sensitivity to salt stress at both vegetative and reproductive phases (Das et al., 2020). Intensive efforts in characterizing the salinity-tolerant Pokkali identified a QTL “*Saltol*” as regulating the uptake of cationic salts into the roots (Das et al., 2020). Since salinity is a major abiotic stress for rice, in the present study, a Pokkalai-derived RIL—namely, FL478—harboring the salinity-tolerant loci “*Saltol*” was used as a donor, and the introgression of “*Salto*l” loci conferred a comparable level of tolerance to salinity in the inter-mated F_7_ progenies of CBMAS 14065. The inter-mated F_7_ progenies of CBMAS 14065 and FL 478 recorded a relatively lesser reduction in their plant height and total dry matter accumulation when compared to that of the recurrent parent CBMAS 14065 and Improved White Ponni (**Table 5**) and the total dry matter production. The above-mentioned results were similar to that of the earlier reports where the *Saltol*-locus-introgressed lines of popular rice varieties exhibited enhanced tolerance to salinity than their recurrent parents (Valarmathi et al., 2019; Singh et al., 2021).

About 12–14 Mha of rice crop in India is frequently affected by flash-flooding submergence, and the discovery of a major effect QTL “*Sub 1*” from flood-tolerant FR 13A paved the way to develop submergence-tolerant versions of popular rice varieties (Neeraja et al., 2007; Rahman et al., 2018). In the present study, efforts were made to pyramid the submergence-tolerant loci “*Sub 1*” from FR13A into CBMAS 14065 along with other biotic/abiotic-stress-tolerant QTLs/genes. All of the inter-mated F_7_ progenies exhibited 90%–96% of recovery after 14 days of submergence stress, whereas the recurrent parent CBMAS 14065 showed 100% susceptibility (**Figure 5**). Relative plant elongation percentage (RPE) was found to be high in the recurrent parent (123.42%) and found to be low in the donor parent and the inter-mated F_7_ progenies (80.69% to 113.84%). The marker-assisted introgression of *Sub1* loci was followed to develop several flood-tolerant rice varieties—namely, CR Dhan 801, Bahuguni dhan-1 and Bahugunidhan-2, Swarna, Samba Mahsuri, TDK *Sub1*, BR 11 *Sub1*, CR 1009 *Sub1*, and CO 43 *Sub1*, which are now extensively cultivated in different parts of India (Septiningsih et al., 2015; Rahman et al., 2018; Sandhu et al., 2019). In a similar manner, the present study has paved the way for the development of submergence-tolerant rice in the genetic background of CBMAS 14065, a derivative of a popular rice variety of South India—namely, Improved White Ponni.

Out of the several blast resistance genes, *Pi9* is reported to exhibit broad-spectrum resistance to different pathotypes of the blast fungus (Liu et al., 2002; Luo et al., 2017). Large-scale testing of rice genotypes harboring different blast resistance genes demonstrated that the efficacy of *Pi9* was sufficient enough to confer a high level of resistance to the most virulent Eastern Indian pathotype of blast fungus (Imam et al., 2014; Variar et al, 2019). Hence, efforts were made to pyramid *Pi9* into CBMAS 14065 along with target QTLs for other biotic/abiotic stresses. The inter-mated progenies of CBMAS 14065 harboring *Pi9* locus exhibited a high level of resistance to a local isolate of the blast pathogen (score = 0) as compared to CBMAS 14065 and the susceptible check CO 39 (Score = 7 to 9) (**Figure 6**). Khanna et al. (2015) had successfully introgressed seven blast genes into Pusa Basmati 1 (PB1), and they discovered that, among the seven genes, the *Pi9-*carrying inter-mated population showed the highest resistance against the virulent races of blast diseases, particularly in hotspot areas. Furthermore, this study has opened up avenues for pyramiding other blast resistance like *Pi54* which will further broaden the genetic base against blast.

The most realistic/practical way of enhancing resistance to bacterial leaf blight pathogen is to combine two or more R genes reported (Mundt et al., 1999). Among the numerous bacterial leaf blight genes reported, the broad-spectrum *Xa21*, in combination with *xa13*, demonstrated broad-spectrum resistance to diverse races of bacterial blight pathogen (Pradhan et al., 2016). In this study, IMF_7_ progenies harboring *xa13* and *Xa21* showed a high level of resistance to local isolate(s) of bacterial blight infection when compared to the recurrent parent CBMAS 14065 (**Figures 7A, B**). The pyramided lines showed significantly lesser lesion length (<0.3 cm) when compared to the recurrent parent. Pyramiding of multiple QTLs/genes conferring resistance to various biotic and abiotic stresses through conventional breeding is difficult and consumes a longer time. Recent advances in marker technology, genotyping, and mating designs provided an opportunity to accelerate the development of multiple-stress-tolerant rice with significant reduction in time, space, labor, and money. In the present study, the development of early generation backcross progenies of CBMAS 14065 harboring different stress-tolerant QTLs and the planned inter-mating of backcross progenies combined with precision genotyping and phenotyping demonstrated the accelerated development of climate-resilient rice requiring less chemicals. Further evaluation of these progenies across environments will identify elite lines exhibiting a consistent performance across environments. These rice genotypes will also serve as genetic stocks for further pyramiding of new traits to enhance their performance and climate resilience.

## Conclusion

The present study clearly demonstrated the success of MAS in combining tolerance to multiple biotic and abiotic stresses while maintaining a higher yield potential and the preferred grain quality. The developed population containing eight distinct QTLs/genes in the current study demonstrated superiority over recurrent parents for multiple abiotic and biotic stresses (drought, submergence, salinity, blast, bacterial leaf blight, and gall midge) as well as for use as genetic stock in future breeding programs.

## Data availability statement

The original contributions presented in the study are included in the article / **Supplementary Material**, Further enquires can be directed to the corresponding Authors.

## Author contributions

RM: Conceptualization, Funding acquisition, Project administration, Supervision, Writing – original draft, Writing – review & editing. VMo: Formal analysis, Investigation, Methodology, Writing – original draft. VMu: Investigation, Methodology, Writing – original draft. RK: Data curation, Software, Writing – original draft. RP: Data curation, Investigation, Writing – original draft. PS: Investigation, Validation, Writing – original draft. BA: Resources, Validation, Writing – original draft. VR: Resources, Supervision, Writing – original draft. SM: Project administration, Resources, Supervision, Writing – review & editing. RR: Methodology, Project administration, Resources, Writing – review & editing. HR: Methodology, Writing – review & editing. JN: Software, Validation, Writing – review & editing. MS: Project administration, Resources, Supervision, Writing – review & editing. GC: Resources, Validation, Writing – review & editing. GV: Project administration, Writing – review & editing.

## References

[B1] Bin A. R. Zhang J. (2023). Trends in rice research: 2030 and beyond. Food Energy Secur. 12, e390. doi: 10.1002/fes3.390

[B2] Das G. Rao G. (2015). Molecular marker assisted gene stacking for biotic and abiotic stress resistance genes in an elite rice cultivar. Front. Plant Sci. 6, 698. doi: 10.3389/fpls.2015.00698 26483798 PMC4588116

[B3] Das G. Rao G. J. Varier M. Prakash A. Prasad D. (2018). Improved Tapaswini having four BB resistance genes pyramided with six genes/QTLs, resistance/tolerance to biotic and abiotic stresses in rice. Sci. Rep. 8. 10.1038/s41598-018-20495-x PMC5799378 29402905

[B4] Divya D. Nair S. Bentur J. (2018). Expression profiles of key genes involved in rice gall midge interactions reveal diversity in resistance pathways. Curr. Sci. 115, 74–82. doi: 10.18520/cs/v115/i1/74-82

[B5] Das S. Kumar A. Barman M. Pal S. Bandopadhyay P. (2020). Impact of climate variability on phenology of rice. Agronomic Crops: Volume 3: Stress Responses Tolerance 13–28. doi: 10.1007/978-981-15-0025-1_2

[B6] Dixit S. Singh U. M. Singh A. K. Alam S. Venkateshwarlu C. Nachimuthu V. V.. (2020). Marker assisted forward breeding to combine multiple biotic-abiotic stress resistance/tolerance in rice. Rice 13, 1–15. doi: 10.1186/s12284-020-00391-7 32472217 PMC7260318

[B7] Ghimire K. H. Quiatchon L. A. Vikram P. Swamy B. M. Dixit S. Ahmed H.. (2012). Identification and mapping of a QTL (qDTY1. 1) with a consistent effect on grain yield under drought. Field Crops Res. 131, 88–96. doi: 10.1016/j.fcr.2012.02.028

[B8] Hasan M. M. Rafii M. Y. Ismail M. R. Mahmood M. Rahim H. A. Alam M. A.. (2015). Marker-assisted backcrossing: a useful method for rice improvement. Biotechnol. Biotechnol. Equip. 29, 237–254. doi: 10.1080/13102818.2014.995920 26019637 PMC4433898

[B9] Imam J. Alam S. Mandal N. P. Variar M. Shukla P. (2014). Molecular screening for identification of blast resistance genes in North East and Eastern Indian rice germplasm (Oryza sativa L.) with PCR based makers. Euphytica 196, 199–211. doi: 10.1007/s10681-013-1024-x

[B10] Janaki Ramayya P. Vinukonda V. P. Singh U. M. Alam S. Venkateshwarlu C. Vipparla A. K.. (2021). Marker-assisted forward and backcross breeding for improvement of elite Indian rice variety Naveen for multiple biotic and abiotic stress tolerance. PloS One 16, e0256721. doi: 10.1371/journal.pone.0256721 34473798 PMC8412243

[B11] Joneydi M. S. (2012). Factors affecting in sustainability of agricultural production systems in Iran. Ann. Biol. Res. 3, 4578–4583.

[B12] Kauffman H. (1973). An improved technique for evaluating resistance of rice varieties to Xanthomonas oryzae. Plant Dis. Rep. 57, 537–541.

[B13] Kaushal L. Ulaganathan K. Shenoy V. Balachandran S. (2018). Geno-and phenotyping of submergence tolerance and elongated uppermost internode traits in doubled haploids of rice. Euphytica 214, 1–16. doi: 10.1007/s10681-018-2305-1

[B14] Khanna A. Sharma V. Ellur R. K. Shikari A. B. Gopala Krishnan S. Singh U.. (2015). Development and evaluation of near-isogenic lines for major blast resistance gene (s) in Basmati rice. Theor. Appl. Genet. 128, 1243–1259. doi: 10.1007/s00122-015-2502-4 25869921

[B15] Khush G. S. Jena K. (2009). Current status and future prospects for research on blast resistance in rice (Oryza sativa L.). Adv. genetics Genomics control Rice blast Dis., 1–10.

[B16] Kumar A. Sandhu N. Dixit S. Yadav S. Swamy B. Shamsudin N. A. A. (2018). Marker-assisted selection strategy to pyramid two or more QTLs for quantitative trait-grain yield under drought. Rice 11, 1–16. doi: 10.1186/s12284-018-0227-0 29845495 PMC5975061

[B17] Liu G. Lu G. Zeng L. Wang G.-L. (2002). Two broad-spectrum blast resistance genes, Pi9 (t) and Pi2 (t), are physically linked on rice chromosome 6. Mol. Genet. Genomics 267, 472–480. doi: 10.1007/s00438-002-0677-2 12111554

[B18] Luo W. Huang M. Guo T. Xiao W. Wang J. Yang G.. (2017). Marker-assisted selection for rice blast resistance genes Pi2 and Pi9 through high-resolution melting of a gene-targeted amplicon. Plant Breed. 136, 67–73. doi: 10.1111/pbr.12447

[B19] Mishra K. K. Vikram P. Yadaw R. B. Swamy B. M. Dixit S. Cruz M. T. S.. (2013). qDTY 12. 1: a locus with a consistent effect on grain yield under drought in rice. BMC Genet. 14, 1–10. doi: 10.1186/1471-2156-14-12 23442150 PMC3616849

[B20] Mundt C. C. Ahmed H. U. Finckh M. R. Nieva L. P. Alfonso R. F. (1999). Primary disease gradients of bacterial blight of rice. Phytopathology 89, 64–67. doi: 10.1094/PHYTO.1999.89.1.64 18944805

[B21] Muthu V. Abbai R. Nallathambi J. Rahman H. Ramasamy S. Kambale R.. (2020). Pyramiding QTLs controlling tolerance against drought, salinity, and submergence in rice through marker assisted breeding. PloS One 15, e0227421. doi: 10.1371/journal.pone.0227421 31910435 PMC6946594

[B22] Nahar S. Sahoo L. Tanti B. (2018). Screening of drought tolerant rice through morpho-physiological and biochemical approaches. Biocatalysis Agric. Biotechnol. 15, 150–159. doi: 10.1016/j.bcab.2018.06.002

[B23] Neeraja C. N. Maghirang-Rodriguez R. Pamplona A. Heuer S. Collard B. C. Septiningsih E. M.. (2007). A marker-assisted backcross approach for developing submergence-tolerant rice cultivars. Theor. Appl. Genet. 115, 767–776. doi: 10.1007/s00122-007-0607-0 17657470

[B24] Pancaldi F. Trindade L. M. (2020). Marginal lands to grow novel bio-based crops: a plant breeding perspective. Front. Plant Sci. 11, 227. doi: 10.3389/fpls.2020.00227 32194604 PMC7062921

[B25] Pandey P. Irulappan V. Bagavathiannan M. V. Senthil-Kumar M. (2017). Impact of combined abiotic and biotic stresses on plant growth and avenues for crop improvement by exploiting physio-morphological traits. Front. Plant Sci. 8, 537. doi: 10.3389/fpls.2017.00537 28458674 PMC5394115

[B26] Pradhan S. K. Nayak D. K. Pandit E. Behera L. Anandan A. Mukherjee A. K.. (2016). Incorporation of bacterial blight resistance genes into lowland rice cultivar through marker-assisted backcross breeding. Phytopathology 106, 710–718. doi: 10.1094/PHYTO-09-15-0226-R 26976728

[B27] Pradhan S. K. Pandit E. Pawar S. Baksh S. Y. Mukherjee A. K. Mohanty S. P. (2019). Development of flash-flood tolerant and durable bacterial blight resistant versions of mega rice variety ‘Swarna’through marker-assisted backcross breeding. Sci. Rep. 9, 12810. doi: 10.1038/s41598-019-49176-z 31488854 PMC6728354

[B28] Qu S. Liu G. Zhou B. Bellizzi M. Zeng L. Dai L.. (2006). The broad-spectrum blast resistance gene Pi9 encodes a nucleotide-binding site–leucine-rich repeat protein and is a member of a multigene family in rice. Genetics 172, 1901–1914. doi: 10.1534/genetics.105.044891 16387888 PMC1456263

[B29] Rahman H. Dakshinamurthi V. Ramasamy S. Manickam S. Kaliyaperumal A. K. Raha S.. (2018). Introgression of submergence tolerance into CO 43, a popular rice variety of India, through marker-assisted backcross breeding. Czech J. Genet. Plant Breed. 54, 101–108. doi: 10.17221/149/2017-CJGPB

[B30] Ramalingam J. Savitha P. Alagarasan G. Saraswathi R. Chandrababu R. (2017). Functional marker assisted improvement of stable cytoplasmic male sterile lines of rice for bacterial blight resistance. Front. Plant Sci. 8. doi: 10.3389/fpls.2017.01131 PMC5489691 28706525

[B31] Rana M. M. Takamatsu T. Baslam M. Kaneko K. Itoh K. Harada N.. (2019). Salt tolerance improvement in rice through efficient SNP marker-assisted selection coupled with speed-breeding. Int. J. Mol. Sci. 20. doi: 10.3390/ijms20102585 PMC6567206 31130712

[B32] Ray D. K. Mueller N. D. West P. C. Foley J. A. (2013). Yield trends are insufficient to double global crop production by 2050. PloS One 8, e66428. doi: 10.1371/journal.pone.0066428 23840465 PMC3686737

[B33] Reay D. Reay D. (2019). Climate-smart rice. Climate-Smart Food, 121–133. doi: 10.1007/978-3-030-18206-9

[B34] Roser M. Ritchie H. Ortiz-Ospina E. Rodés-Guirao L. (2013). World population growth. Our World data. Available at: https://ourworldindata.org/worldpopulation-growth.

[B35] Sandhu N. Dixit S. Swamy B. Raman A. Kumar S. Singh S.. (2019). Marker assisted breeding to develop multiple stress tolerant varieties for flood and drought prone areas. Rice 12, 1–16. doi: 10.1186/s12284-019-0269-y 30778782 PMC6379507

[B36] Septiningsih E. M. Hidayatun N. Sanchez D. L. Nugraha Y. Carandang J. Pamplona A. M.. (2015). Accelerating the development of new submergence tolerant rice varieties: the case of Ciherang-Sub1 and PSB Rc18-Sub1. Euphytica 202, 259–268. doi: 10.1007/s10681-014-1287-x

[B37] Singh G. Kaur N. Khanna R. Kaur R. Gudi S. Kaur R.. (2024). 2Gs and plant architecture: breaking grain yield ceiling through breeding approaches for next wave of revolution in rice (Oryza sativa L.). Crit. Rev. Biotechnol. 44, 139–162. doi: 10.1080/07388551.2022.2112648 36176065

[B38] Singh V. J. Vinod K. K. Krishnan S. G. Singh A. K. (2021). Rice adaptation to climate change: opportunities and priorities in molecular breeding. Mol. Breed. Rice abiotic Stress tolerance Nutr. Qual., 1–25. doi: 10.1002/9781119633174.ch1

[B39] Singh V. K. Singh B. D. Kumar A. Maurya S. Krishnan S. G. Vinod K. K.. (2018). Marker-assisted introgression of Saltol QTL enhances seedling stage salt tolerance in the rice variety “Pusa Basmati 1. Int. J. Genomics 2018. doi: 10.1155/2018/8319879 PMC5896403 29785398

[B40] Sultana H. Somaddar U. Samanta S. C. Chowdhury A. K. Saha G. (2022). Diversity analysis of Bangladeshi coastal rice landraces (Oryza sativa) for morpho-physiological and molecular markers’ responses to seedling salinity tolerance. Plant Breed. Biotechnol. 10, 115–127. doi: 10.9787/PBB.2022.10.2.115

[B41] Tiwari S. Tomar R. Tripathi M. Ahuja A. (2017). Modified protocol for plant genomic DNA isolation. Indian Res. J. Genet. Biotechnol. 9, 478–485.

[B42] Valarmathi M. Sasikala R. Rahman H. Jagadeeshselvam N. Kambale R. Raveendran M. (2019). Development of salinity tolerant version of a popular rice variety improved white ponni through marker assisted back cross breeding. Plant Physiol. Rep. 24, 262–271. doi: 10.1007/s40502-019-0440-x

[B43] Variar M. Cruz C. V. Carrillo M. Bhatt J. Sangar R. (2019). “Rice blast in India and strategies to develop durably resistant cultivars,” in Advances in genetics, genomics and control of rice blast disease (Netherlands: Springer), 359–374.

[B44] Venuprasad R. Dalid C. Del Valle M. Zhao D. Espiritu M. Sta Cruz M.. (2009). Identification and characterization of large-effect quantitative trait loci for grain yield under lowland drought stress in rice using bulk-segregant analysis. Theor. Appl. Genet. 120, 177–190. doi: 10.1007/s00122-009-1168-1 19841886

[B45] Vikram P. Swamy B. M. Dixit S. Ahmed H. U. Teresa Sta Cruz M. Singh A. K.. (2011). qDTY 1.1, a major QTL for rice grain yield under reproductive-stage drought stress with a consistent effect in multiple elite genetic backgrounds. BMC Genet. 12, 1–15. doi: 10.1186/1471-2156-12-89 22008150 PMC3234187

[B46] Vikram P. Swamy B. M. Dixit S. Singh R. Singh B. P. Miro B.. (2015). Drought susceptibility of modern rice varieties: an effect of linkage of drought tolerance with undesirable traits. Sci. Rep. 5, 14799. doi: 10.1038/srep14799 26458744 PMC4602206

[B47] Wu J. Kim S. G. Kang K. Y. Kim J.-G. Park S.-R. Gupta R.. (2016). Overexpression of a pathogenesis-related protein 10 enhances biotic and abiotic stress tolerance in rice. Plant Pathol. J. 32, 552. doi: 10.5423/PPJ.OA.06.2016.0141 27904462 PMC5117864

[B48] Yamori W. (2013). Improving photosynthesis to increase food and fuel production by biotechnological strategies in crops. J. Plant Biochem. Physiol. 1. doi: 10.4172/2329-9029.1000113

[B49] Yasmin S. Hafeez F. Y. Mirza M. S. Rasul M. Arshad H. M. Zubair M.. (2017)Biocontrol of bacterial leaf blight of rice and profiling of secondary metabolites produced by rhizospheric Pseudomonas aeruginosa BRp3. Front. Microbiol. 8. doi: 10.3389/fmicb.2017.01895 PMC5622989 29018437

[B50] Yoshida S. (1976). Laboratory manual for physiological studies of rice. Int. Rice Res. Ins Philippines 23, 61–66.

[B51] Yugander A. Sundaram R. M. Singh K. Ladhalakshmi D. Subba Rao L. V. Madhav M. S.. (2018). Incorporation of the novel bacterial blight resistance gene Xa38 into the genetic background of elite rice variety Improved Samba Mahsuri. PloS One 13, e0198260. doi: 10.1371/journal.pone.0198260 29813124 PMC5973576

[B52] Zheng W. Wang Y. Wang L. Ma Z. Zhao J. Wang P.. (2016). Genetic mapping and molecular marker development for Pi65 (t), a novel broad-spectrum resistance gene to rice blast using next-generation sequencing. Theor. Appl. Genet. 129, 1035–1044. doi: 10.1007/s00122-016-2681-7 26883042

