# Supplementary material for: Marker-assisted breeding accelerates the development of multiple-stress-tolerant rice genotypes adapted to wider environments
Source: Front Plant Sci. 2024 Jul 12;15:1402368. doi: 10.3389/fpls.2024.1402368 (PMC11272538; doi:10.3389/fpls.2024.1402368)
Supplement: Supplementary file 1 [file Table_1.docx]

**Supplementary Table 1. List of important QTLs/genes linked to major biotic / abiotic stress tolerance traits in rice**

| **QTLs** | **Trait** | **Donor Lines** | **Reference** |
| --- | --- | --- | --- |
| **Drought Tolerance** | | | |
| *qDTY1.1* | Grain yield under drought | Apo, N22, Dhagaddeshi, CT9993-10-1-M, Kali Aus, Basmati 334 | (Kumar et al., 2008; Vikram et al., 2011; Ghimire et al., 2012; Sandhu et al., 2014) |
| *qDTY1.2* |  | Kali Aus | (Sandhu et al., 2014) |
| *qDTY1.3* |  | Kali Aus | (Sandhu et al., 2014) |
| *qDTY2.1* |  | Apo, Aus 276 | (Venuprasad et al., 2009; Sandhu et al., 2014) |
| *qDTY2.2* |  | Aday Sel, Kali Aus | (Swamy and Kumar, 2013; Palanog et al., 2014; Sandhu et al., 2014) |
| *qDTY2.3* |  | Kali Aus | (Palanog et al., 2014; Sandhu et al., 2014) |
| *qDTY3.1* |  | Apo, IR55419-04 | (Venuprasad et al., 2009; Dixit et al., 2014) |
| *qDTY3.2* |  | N22, IR77298-5-6-18, Aday sel | (Pidgeon et al., 2006; Vikram et al., 2011) |
| *qDTY4.1* |  | Aday Sel | (Swamy and Kumar, 2013) |
| *qDTY6.1* |  | Apo, Vandana, IR55419-04 | (Venuprasad et al., 2012; Dixit et al., 2014) |
| *qDTY6.2* |  | IR55419-04 | (Dixit et al., 2014) |
| *qDTY9.1* |  | Aday sel | (Swamy and Kumar, 2013) |
| *qDTY10.1* |  | N22, Aday sel, Basmati 334 | (Vikram et al., 2011; Swamy and Kumar, 2013) |
| *qDTY10.2* |  | Aday sel | (Swamy and Kumar, 2013) |
| *qDTY12.1* |  | Way Rarem, IR74371-46-1-1 | (Bernier et al., 2009; Mishra et al., 2013) |
| *qLR9.1* | Leaf rolling | CR 143-2-2 | (Barik et al., 2019) |
| *qLR8.1* | Leaf rolling | CR 143-2-2 | (Barik et al., 2019) |
| *qLD9.1* | Leaf drying | CR 143-2-2 | (Barik et al., 2019) |
| *qLD12.1* | Leaf drying | CR 143-2-2 | (Barik et al., 2019) |
| *qHI9.1* | Harvest index | CR 143-2-2 | (Barik et al., 2019) |
| *qSF9.1* | Spikelet fertility | CR 143-2-2 | (Barik et al., 2019) |
| *qRWC9.1* | RWC | CR 143-2-2 | (Barik et al., 2019) |
| *qgy3.1* | Panicle number | CT9993-510-1-M | (Lanceras et al., 2004) |
| *qPH1.1* | Plant height | Cabacu (IRAT177) | (Trijatmiko et al., 2014) |
| *qHGW2.2* | 100 grain weight | Moroberekan | (Sellamuthu et al., 2015) |
| *qGy7* | Grain weight | Lemont | (Zhao et al., 2008) |
| *qPL-9* | Panicle length | IRAT109 | (Liu et al., 2008) |
| *qDTY8.1* | Grain number | Basmati 334 | (Vikram et al., 2012) |
| *qSf6* | spikelet fertility | Tarom Molaei | (Wang et al., 2013) |
| *qPNF3.1* | Grain yield | Moroberekan | (Sellamuthu et al., 2015) |
| *qPDL1.2* | Grain yield | Moroberekan | (Sellamuthu et al., 2015) |
| *qPSS8.1* | Percent seed set | Cabacu (IRAT 177) | (Trijatmiko et al., 2014) |
| **Salinity Tolerance** | | | |
| *Saltol* | seedling stage salt tolerance | FL478 (pokkali) | (Singh et al., 2018) |
| *QTL1* | Seedling stage | FL478 (pokkali) | (Ammar et al., 2007) |
| *qSTR6* | Seedling stage | Tarommahali | (Sabouri et al., 2009) |
| *qST1 and qST3* | young seedling stage | RILs (‘Milyang 23’ (indica) x ‘Gihobyeo’ (japonica)) | (Lee et al., 2007) |
| *qSKC-1* | Shoot K^+^ concentration | Nona Bokra (*indica*)/ Koshihikari (*japonica*) | (Jing et al., 2017) |
| **Submergence Tolerance** | | | |
| *SUB1A* | Flash flooding | FR13A | (Oladosu et al., 2020) |
| *SK1, SK2* | Deep water flooding | *Oryza rufipogon, Oryza nivara*, and *Oryza glumaepatula* | (Sasayama et al., 2018) |
| *SUB1B, SUB1C* | plant quiescence | *O. sativa* accessions | (Fukao and Bailey-Serres, 2008) |
| **Blast** | | | |
| *Pi37* | NBS-LRR | St. No. 1 | (Lin et al., 2007) |
| *Pit* | CC-NBS-LRR | K59 | (Hayashi and Yoshida, 2009) |
| *Pish* | NBS-LRR | Shin-2 | (Takahashi et al., 2010) |
| *Pi35 #* | NBS-LRR | Hokkai 188 | (Fukuoka et al., 2014) |
| *Pi64* | NBS-LRR | Yangmaogu | (Ma et al., 2015) |
| *Pib* | NBS-LRR | Tohoku IL9 | (Wang et al., 1999) |
| *pi21 #* | Proline-rich metal binding protein | Owarihatamochi | (Fukuoka et al., 2009) |
| *Pi63/Pikahei-1(t) #* | NBS-LRR | Kahei | (Xu et al., 2014) |
| *Pi-d2* | B-lectin receptor kinase | Digu | (Chen et al., 2006) |
| *Pi9* | NBS-LRR | 75-1-127 | (Qu et al., 2006) |
| *Pi2* | NBS-LRR | C101A51 | (Zhou et al., 2006) |
| *Piz-t* | NBS-LRR | Toride 1 | (Zhou et al., 2006) |
| *Pi-d3* | CC-NBS-LRR | Digu | (Shang et al., 2009) |
| *Pi25* | CC-NBS-LRR | Gumei 2 | (Chen et al., 2011) |
| *Pi50* | NBS-LRR | Er-Ba-zhan (EBZ) | (Su et al., 2015) |
| *Pid3-I1* | CC-NBS-LRR | MC276 | (Inukai et al., 2019) |
| *Pi36* | CC-NBS-LRR | Q61 | (Liu et al., 2007) |
| *Pi5* | CC-NBS-LRR | Moroberekan | (Lee et al., 2009) |
| *Pii* | NBS-LRR | Hitomebore | (Takagi et al., 2013) |
| *Pikm* | NBS-LRR | Tsuyuake | (Ashikawa et al., 2008) |
| *Pb1 #* | CC-NBS-LRR | Modan | (Hayashi et al., 2010) |
| *Pi54* | NBS-LRR | Tetep | (Sharma et al., 2010) |
| *Pia* | CC-NBS-LRR | Aichi Asahi | (Okuyama et al., 2011) |
| *Pik-p* | CC-NBS-LRR | K60 | (Yuan et al., 2011) |
| *Pik* | CC-NBS-LRR | Kusabue | (Zhai et al., 2011) |
| *Pi1* | NBS-LRR | C101LAC | (Hua et al., 2012) |
| *Pi-CO39* | CC-NBS-LRR | CO39 | (Cesari et al., 2013) |
| *Pike* | NBS-LRR | Xiangzao 143 | (Chen et al., 2015) |
| *Pi-ta* | NBS-LRR | Yashiro-mochi | (Bryan et al., 2000) |
| *Ptr* | Atypical protein with an armadillo repeat | Katy | (Zhao et al., 2018) |
| **Bacterial Leaf Blight** | | | |
| *Xa1* | Dominant | Kogyoku, Java 14 | (Sakaguchi, 1967; Yoshimura et al., 1998) |
| *Xa2* | Dominant | RantaiEmas II, Tetep | (Sakaguchi, 1967; He et al., 2006) |
| *Xa3/Xa26* | Dominant | Wase Aikoku 3 | (Ezuka, 1975; Yoshimura et al., 1992; Gao et al., 2013) |
| *Xa4* | Dominant | TKM6, IR20, IR22, IR72 | (Petpisit et al., 1977) |
| *xa5* | Recessive | DZ192, IR1545-339 | (Petpisit et al., 1977; Blair et al., 2003) |
| *Xa6/xa3* | Dominant | MalagkitSungsong Zenith | (Sidhu et al., 1978) |
| *Xa7* | Dominant | DZ78, DV85 | (Sidhu et al., 1978) |
| *xa8* | Recessive | PI231129 | (Singh et al., 2002) |
| *Xa9* | Dominant | KhaolayNhay | (Singh et al., 1983; Ogawa and Khush, 1989) |
| *Xa10* | Dominant | Cas 209 | (Yoshimura et al., 1983) |
| *Xa11* | Dominant | RP9-3 | (Ogawa and Yamamoto, 1986; Goto et al., 2009) |
| *Xa12* | Dominant | Kogyoku, Java 14 | (Ogawa et al., 1978) |
| *xa13* | Recessive | BJ1, ChinsurahEoro II | (Yoshimura et al., 1995; Zhang et al., 1996) |
| *Xa14* | Dominant | TN1 | (Taura et al., 1992) |
| *xa15* | Recessive | M41 | (Nakai et al., 1988) |
| *Xa16* | Dominant | Tetep | (Sanchez et al., 1999) |
| *Xa17* | Dominant | Asominori | (Ogawa and Khush, 1989) |
| *Xa18* | Dominant | Toyonishiki, Milyang 23, IR24 | (Ogawa and Yamamoto, 1986) |
| *xa19* | Recessive | XM5 | (Taura et al., 1992) |
| *xa20* | Recessive | XM6 | (Taura et al., 1992) |
| *Xa21* | Dominant | O. longistaminata | (Khush et al., 1990) |
| *Xa22(t)* | Dominant | Zhachanglong | (Lin et al., 1996) |
| *Xa23* | Dominant | O. rufipogon | (Zhang et al., 1998) |
| *xa24* | Recessive | DV86, DV85, Aus 295 | (Khush et al., 1990) |
| *xa25(t)* | Recessive | HX-3, Minghui 63 | (Lee et al., 2003) |
| *xa26(t)* | Recessive | Minghui 63 | (Lee et al., 2003) |
| *Xa27(t)* | Dominant | O. minuta IRGC101141,  IRBB27 | (Amante-Bordeos et al., 1992; Lee et al., 2003; Gu et al., 2004) |
| *xa28(t)* | Recessive | Lota Sail | (Lee et al., 2003) |
| *Xa29(t)* | Dominant | B5 | (Tan et al., 2004) |
| *Xa30(t)* | Dominant | O. rufipogon Y238 | (Xuwei et al., 2007; Cheema et al., 2008) |
| *xa31(t)* | Recessive | Zhachanglong | (Wang et al., 2009) |
| *xa32(t)* | Recessive | C4064 | (Zheng et al., 2009) |
| *Xa33* | Dominant | *O. nivara* | (Kumar et al., 2012) |
| *xa34(t)* | Recessive | *O. brachyantha* | (Ram et al., 2010) |
| *Xa35(t)* | Dominant | *O. minuta* | (Guo et al., 2010) |
| *Xa36(t)* | Dominant | C4059 | (Miao et al., 2010) |
| *Xa38* | Dominant | *O. nivara* IRGC81825 | (Cheema et al., 2008) |
| *Xa39* | Dominant | FF329 | (Zhang et al., 2015) |
| *Xa40(t)* | Dominant | IR65482-7-216-1-2 | (Kim et al., 2015) |
| *xa41(t)* | Recessive | Rice germplasm | (Hutin et al., 2015) |
| *xa42* | Recessive | XM14, a mutant of IR24 | (Busungu et al., 2018) |
| *Xa43(t)* | Dominant | P8 | (Kim and Reinke, 2019) |
| *xa44(t)* | Recessive | IR73571-3B-11-3-K3 | (Kim, 2018) |
| *xa45(t)* | Recessive | *O. glaberrima* IRGC102600B | (Neelam et al., 2020) |
| *xa46(t)* | Recessive | Mutant H120 | (Chen et al., 2020) |
| **Gall midge** | | | |
| *Gm 1* | R gene(HR- type) | Kavya | (Biradar et al., 2004) |
| *Gm 2* | R gene(HR+ type) | Phalguna | (Mohan et al., 1994) |
| *gm 3* | R gene (HR+ type) | RP 2068-18-3-5 | (Katiyar et al., 2000) |
| *Gm 4* | R gene (HR+ type) | Abhaya | (Nanda et al., 2010) |
| *Gm 5* | R gene (HR+ type) | ARC5984 | (Lima et al., 2007) |
| *Gm 6(t)* | R gene (HR+ type) | Duokang 1 | (Katiyar et al., 2001) |
| *Gm 7* | R gene (HR+ type) | RP2333-156-8 | (Jain et al., 2004) |
| *Gm 8* | R gene(HR- type) | Jhitpiti/Aganni | (Sardesai et al., 2002; Jain et al., 2004) |
| *Gm 9* | R gene (HR+ type) | Madhuri Line 9 | (Shrivastava et al., 2003) |
| *Gm 10* | R gene (HR+ type) | BG-380-2 | (Kumar et al., 2005) |
| *Gm 11* | R gene (HR+ type) | CR57-MR1523 | (Himabindu et al., 2010) |

**Supplementary Table 2. Improved genetic stocks of rice pyramided with multiple QTLs / genes through MABB**

| **Recipient Parent** | **Target Trait(s)** | **QTLs/genes linked** | **Reference** |
| --- | --- | --- | --- |
| Swarna | Resistance to Blast, Blight, Gall midge and Drought tolerance | *Pi9, Xa4, xa5, xa13, Xa21, Bph3, Bph17, Gm4, Gm8, qDTY1.1 and qDTY3.1* | (Dixit et al., 2020) |
| Improved Lalat | Resistance to Blast, Gall midge and BLB; Tolerance to salinity and submergence | *Pi2,Pi9*, *Gm1, Gm4*, *Sub1*, *Saltol*, *xa5*, *xa13,* and *Xa21* | (Das and Rao, 2015) |
| Lalat | Drought tolerance, Blast and Blight resistance | *qDTY1.1, qDTY3.1, qDTY12.1, Xa4, xa5, xa13, Xa21 and Pi9* | (Singh et al., 2022) |
| Pink3 | Submergence tolerance; resistance to BLB, Blast and BPH | *Sub1A-C, SSIIa, Xa5,*  *Xa21, TPS, qBph3,*  *qBL1, qBL11* | (Ruengphayak et al., 2015) |
| CB 87 R and CB 174 R | Resistance to BLB, Blast and Sheath blight | *xa5, xa13, Xa21, Pi54, qSBR7-1, qSBR11-1, and qSBR11-2* | (Ramalingam et al., 2020) |
| Jumam | Resistance to BPH, Stripe virus, blast and BLB | *Bph18, qSTV11SG,*  *Pib, Pi, xa3, xa40* | (Reinke et al., 2018) |
| Naveen | Resistance to Blast, BLB and Gall midge; Drought tolerance | *Pi9*, *Xa21, Gm8,* qDTY*1*.*1*, *qDTY2*.*2* and *qDTY4*.*1* | (Janaki Ramayya et al., 2021) |
| Swarna | Yield, Submergence tolerance and BLB resistance | *Sub1, xa5, Xa21, SCM2,*  *OsSPL14* | (Mohapatra et al., 2021) |
| RPHR-1005 | Blight and gall  midge resistance | *Xa21, Gm4, Gm8,*  *Rf3, Rf4* | (Abhilash Kumar et al., 2017) |
| Jinbubyeo | Resistance to Blast, BLB and BPH | *Pi40, xa4, xa5,*  *xa21, Bph18* | (Kottapalli et al., 2010) |
| Tellahamsa | Resistance to BLB and Blast | *Xa21*, xa*13, Pi54* and *Pi1* | (Jamaloddin et al., 2020) |
| Improved White Ponni | Tolerance to drought, salinity and submergence | *qDTY1*.*1*, *qDTY2*.*1*, *Saltol*, and *Sub1* | (Muthu et al., 2020) |
| Improved Samba Mahsuri | Resistance to Blast and BLB | *Pi2 or Pi54 along with Xa21, xa13 and xa5* | (Rekha et al., 2018) |
| Ranidhan | Bacterial blight resistance and Submergence tolerance | *Xa21, xa13, xa5 and Sub1* | (Mohapatra et al., 2023) |
| Tapaswini | Bacterial blight resistance | *Xa21, xa13, xa5 and Xa4* | (Dokku et al., 2013) |
| Swarna-Sub1 | Drought and  submergence tolerance | *qDTY1.1, qDTY2.1,*  *qDTY3.1, Sub1* | (Sandhu et al., 2019) |
| 9311 | Resistance to Blast and BLB; submergence tolerance | *Pi9, Sub1A, xa21, xa27* | (Luo et al., 2017) |
| WH421 | Resistance to BLB, Blast; submergence tolerance and aroma | *xa27, Pi9,*  *Sub1A, Badh2.1* | (Luo et al., 2016) |
| Pusa Basmati | Blight resistance;  blast resistance | *xa13, xa21, Pi54, Piz-5* | (Singh et al., 2011) |
| Hua-jing-xian 74 | Cold tolerance | *qCTBB-5, qCTBB-6,*  *qCTS-6, qCTS12* | (Yang et al., 2016) |
| NLR3449 | Bacterial blight resistance | *Xa21, xa13 and xa5* | (Dasari et al., 2022) |
| IR9784-226-335-1-5-1-1 | Tolerance to drought and submergence | *qDTY3.1,*  *qDTY12.1, Sub2* | (Mohd Ikmal et al., 2021) |

**Supplementary Table 3. IRRI - Standard Evaluation Score (SES) for blast screening, 2002**

| **Scale** | **Damage** | **Description** |
| --- | --- | --- |
| 0 | No Symptoms | Highly Resistant |
| 1 | Small Brown Specks of Pin point size | Resistant |
| 3 | Infecting less than 4% of leaf area | Moderately Resistant |
| 5 | Infecting less than 4-10% of leaf area | Moderately susceptible |
| 7 | Infecting less than 26-50% of leaf area | Susceptible |
| 9 | Infecting more than 75% of leaf area | Highly susceptible |

**Supplementary Table 4. Scoring of bacterial leaf blight**

| **Green House Test** | |
| --- | --- |
| **Lesion Length (cm)** | **Description** |
| 0-5 | Resistance |
| 5-10 | Moderately Resistance |
| 10-15 | Moderately Susceptible |
| Above 15 | Susceptible |

**Supplementary Table 5. Standard Evaluation System (SES) for measuring salinity**

**tolerance in rice (**[**IRRI 1996**](#_ENREF_4)**)**

| **Score** | **Observation** | **Tolerance** |
| --- | --- | --- |
| 1 | Normal growth, no leaf rolling symptoms | Highly tolerant |
| 3 | Leaf tip drying and leaf rolling symptom | Tolerant |
| 5 | Growth severely retarded; most leaves rolled; only a few are elongating | Moderately tolerant |
| 7 | Complete cessation of growth; most leaves dry; some plants dying | Susceptible |
| 9 | Almost all plants dead or dying | Highly susceptible |

**References**

Abhilash Kumar, V., Balachiranjeevi, C., Bhaskar Naik, S., Rekha, G., Rambabu, R., Harika, G., et al. (2017). Marker-assisted pyramiding of bacterial blight and gall midge resistance genes into RPHR-1005, the restorer line of the popular rice hybrid DRRH-3. *Molecular Breeding* 37**,** 1-14.

Amante-Bordeos, A., Sitch, L., Nelson, R., Dalmacio, R., Oliva, N., Aswidinnoor, H., et al. (1992). Transfer of bacterial blight and blast resistance from the tetraploid wild rice Oryza minuta to cultivated rice, Oryza sativa. *Theoretical and Applied Genetics* 84**,** 345-354.

Ammar, M., Singh, R., Singh, A., Mohapatra, T., Sharma, T., and Singh, N. (Year). "Mapping QTLs for salinity tolerance at seedling stage in rice (Oryza sativa L.)", in: *African crop science conference proceedings*), 617-620.

Ashikawa, I., Hayashi, N., Yamane, H., Kanamori, H., Wu, J., Matsumoto, T., et al. (2008). Two adjacent nucleotide-binding site–leucine-rich repeat class genes are required to confer Pikm-specific rice blast resistance. *Genetics* 180(4)**,** 2267-2276.

Barik, S.R., Pandit, E., Pradhan, S.K., Mohanty, S.P., and Mohapatra, T. (2019). Genetic mapping of morpho-physiological traits involved during reproductive stage drought tolerance in rice. *PLoS One* 14(12)**,** e0214979.

Bernier, J., Serraj, R., Kumar, A., Venuprasad, R., Impa, S., RP, V.G., et al. (2009). The large-effect drought-resistance QTL qtl12. 1 increases water uptake in upland rice. *Field Crops Research* 110(2)**,** 139-146.

Biradar, S., Sundaram, R., Thirumurugan, T., Bentur, J., Amudhan, S., Shenoy, V., et al. (2004). Identification of flanking SSR markers for a major rice gall midge resistance gene Gm1 and their validation. *Theoretical and applied genetics* 109**,** 1468-1473.

Blair, M.W., Garris, A.J., Iyer, A.S., Chapman, B., Kresovich, S., and McCouch, S.R. (2003). High resolution genetic mapping and candidate gene identification at the xa5 locus for bacterial blight resistance in rice (Oryza sativa L.). *Theoretical and Applied Genetics* 107**,** 62-73.

Bryan, G.T., Wu, K.-S., Farrall, L., Jia, Y., Hershey, H.P., McAdams, S.A., et al. (2000). A single amino acid difference distinguishes resistant and susceptible alleles of the rice blast resistance gene Pi-ta. *The Plant Cell* 12(11)**,** 2033-2045.

Busungu, C., Taura, S., Sakagami, J.-I., Anai, T., and Ichitani, K. (2018). High-resolution mapping and characterization of xa42, a resistance gene against multiple Xanthomonas oryzae pv. oryzae races in rice (Oryza sativa L.). *Breeding science* 68(2)**,** 188-199.

Cesari, S., Thilliez, G., Ribot, C., Chalvon, V., Michel, C., Jauneau, A., et al. (2013). The rice resistance protein pair RGA4/RGA5 recognizes the Magnaporthe oryzae effectors AVR-Pia and AVR1-CO39 by direct binding. *The Plant Cell* 25(4)**,** 1463-1481.

Cheema, K.K., Grewal, N.K., Vikal, Y., Sharma, R., Lore, J.S., Das, A., et al. (2008). A novel bacterial blight resistance gene from Oryza nivara mapped to 38 kb region on chromosome 4L and transferred to Oryza sativa L. *Genetics research* 90(5)**,** 397-407.

Chen, J., Shi, Y., Liu, W., Chai, R., Fu, Y., Zhuang, J., et al. (2011). A Pid3 allele from rice cultivar Gumei2 confers resistance to Magnaporthe oryzae. *Journal of Genetics and Genomics* 38(5)**,** 209-216.

Chen, S., Wang, C., Yang, J., Chen, B., Wang, W., Su, J., et al. (2020). Identification of the novel bacterial blight resistance gene Xa46 (t) by mapping and expression analysis of the rice mutant H120. *Scientific reports* 10(1)**,** 12642.

Chen, X., Shang, J., Chen, D., Lei, C., Zou, Y., Zhai, W., et al. (2006). AB‐lectin receptor kinase gene conferring rice blast resistance. *The Plant Journal* 46(5)**,** 794-804.

Chen, Y., Ye, W., Zhang, Y., and Xu, Y. (2015). High speed BLASTN: an accelerated MegaBLAST search tool. *Nucleic acids research* 43(16)**,** 7762-7768.

Das, G., and Rao, G. (2015). Molecular marker assisted gene stacking for biotic and abiotic stress resistance genes in an elite rice cultivar. *Frontiers in plant science* 6**,** 698.

Dasari, A., Vemulapalli, P., Gonuguntla, R., Thota, D.K., Elumalai, P., Muppavarapu, K., et al. (2022). Improvement of bacterial blight resistance of the popular variety, Nellore Mahsuri (NLR34449) through marker-assisted breeding. *Journal of Genetics* 101**,** 1-11.

Dixit, S., Singh, A., Sta Cruz, M.T., Maturan, P.T., Amante, M., and Kumar, A. (2014). Multiple major QTL lead to stable yield performance of rice cultivars across varying drought intensities. *Bmc Genetics* 15**,** 1-13.

Dixit, S., Singh, U.M., Singh, A.K., Alam, S., Venkateshwarlu, C., Nachimuthu, V.V., et al. (2020). Marker assisted forward breeding to combine multiple biotic-abiotic stress resistance/tolerance in rice. *Rice* 13**,** 1-15.

Dokku, P., Das, K., and Rao, G. (2013). Pyramiding of four resistance genes of bacterial blight in Tapaswini, an elite rice cultivar, through marker-assisted selection. *Euphytica* 192**,** 87-96.

Ezuka, A. (1975). Inheritance of resistance of rice variety Wase Aikoku 3 to Xanthomonas oryzae. *Bull. Tokai-Kinki Natl. Agric. Exp. Stn.* 28**,** 124-130.

Fukao, T., and Bailey-Serres, J. (2008). Submergence tolerance conferred by Sub1A is mediated by SLR1 and SLRL1 restriction of gibberellin responses in rice. *Proceedings of the National Academy of Sciences* 105(43)**,** 16814-16819.

Fukuoka, S., Saka, N., Koga, H., Ono, K., Shimizu, T., Ebana, K., et al. (2009). Loss of function of a proline-containing protein confers durable disease resistance in rice. *Science* 325(5943)**,** 998-1001.

Fukuoka, S., Yamamoto, S.-I., Mizobuchi, R., Yamanouchi, U., Ono, K., Kitazawa, N., et al. (2014). Multiple functional polymorphisms in a single disease resistance gene in rice enhance durable resistance to blast. *Scientific Reports* 4(1)**,** 4550.

Gao, L., Tu, Z.J., Millett, B.P., and Bradeen, J.M. (2013). Insights into organ-specific pathogen defense responses in plants: RNA-seq analysis of potato tuber-Phytophthora infestans interactions. *BMC genomics* 14**,** 1-12.

Ghimire, K.H., Quiatchon, L.A., Vikram, P., Swamy, B.M., Dixit, S., Ahmed, H., et al. (2012). Identification and mapping of a QTL (qDTY1. 1) with a consistent effect on grain yield under drought. *Field Crops Research* 131**,** 88-96.

Goto, T., Matsumoto, T., Furuya, N., Tsuchiya, K., and Yoshimura, A. (2009). Mapping of bacterial blight resistance gene Xa11 on rice chromosome 3. *Japan Agricultural Research Quarterly: JARQ* 43(3)**,** 221-225.

Gu, K., Tian, D., Yang, F., Wu, L., Sreekala, C., Wang, D., et al. (2004). High-resolution genetic mapping of Xa27 (t), a new bacterial blight resistance gene in rice, Oryza sativa L. *Theoretical and Applied Genetics* 108**,** 800-807.

Guo, S., Zhang, D., and Lin, X. (2010). Identification and mapping of a novel bacterial blight resistance gene Xa35 (t) originated from Oryza minuta. *Scientia Agricultura Sinica* 43(13)**,** 2611-2618.

Hayashi, K., and Yoshida, H. (2009). Refunctionalization of the ancient rice blast disease resistance gene Pit by the recruitment of a retrotransposon as a promoter. *The Plant Journal* 57(3)**,** 413-425.

Hayashi, N., Inoue, H., Kato, T., Funao, T., Shirota, M., Shimizu, T., et al. (2010). Durable panicle blast‐resistance gene Pb1 encodes an atypical CC‐NBS‐LRR protein and was generated by acquiring a promoter through local genome duplication. *The Plant Journal* 64(3)**,** 498-510.

He, Q., Li, D., Zhu, Y., Tan, M., Zhang, D., and Lin, X. (2006). Fine mapping of Xa2, a bacterial blight resistance gene in rice. *Molecular Breeding* 17**,** 1-6.

Himabindu, K., Suneetha, K., Sama, V., and Bentur, J. (2010). A new rice gall midge resistance gene in the breeding line CR57-MR1523, mapping with flanking markers and development of NILs. *Euphytica* 174**,** 179-187.

Hua, L., Wu, J., Chen, C., Wu, W., He, X., Lin, F., et al. (2012). The isolation of Pi1, an allele at the Pik locus which confers broad spectrum resistance to rice blast. *Theoretical and applied genetics* 125**,** 1047-1055.

Hutin, M., Sabot, F., Ghesquière, A., Koebnik, R., and Szurek, B. (2015). A knowledge‐based molecular screen uncovers a broad‐spectrum Os SWEET 14 resistance allele to bacterial blight from wild rice. *The Plant Journal* 84(4)**,** 694-703.

Inukai, T., Nagashima, S., and Kato, M. (2019). Pid3-I1 is a race-specific partial-resistance allele at the Pid3 blast resistance locus in rice. *Theoretical and Applied Genetics* 132**,** 395-404.

Jain, A., Ariyadasa, R., Kumar, A., Srivastava, M., Mohan, M., and Nair, S. (2004). Tagging and mapping of a rice gall midge resistance gene, Gm8, and development of SCARs for use in marker-aided selection and gene pyramiding. *Theoretical and applied genetics* 109**,** 1377-1384.

Jamaloddin, M., Durga Rani, C.V., Swathi, G., Anuradha, C., Vanisri, S., Rajan, C., et al. (2020). Marker Assisted Gene Pyramiding (MAGP) for bacterial blight and blast resistance into mega rice variety “Tellahamsa”. *PloS one* 15(6)**,** e0234088.

Janaki Ramayya, P., Vinukonda, V.P., Singh, U.M., Alam, S., Venkateshwarlu, C., Vipparla, A.K., et al. (2021). Marker-assisted forward and backcross breeding for improvement of elite Indian rice variety Naveen for multiple biotic and abiotic stress tolerance. *PLoS One* 16(9)**,** e0256721.

Jing, W., Deng, P., Cao, C., and Zhang, W. (2017). Fine mapping of qSKC-1, a major quantitative trait locus for shoot K+ concentration, in rice seedlings grown under salt stress. *Breeding science* 67(3)**,** 286-295.

Katiyar, S., Tan, Y., Huang, B., Chandel, G., Xu, Y., Zhang, Y., et al. (2001). Molecular mapping of gene Gm-6 (t) which confers resistance against four biotypes of Asian rice gall midge in China. *Theoretical and Applied Genetics* 103**,** 953-961.

Katiyar, S., Verulkar, S., Adsul, G., Dhundre, M., Chandel, G., and Bennett, J. (Year). "Molecular markers for gall midge resistance genes in rice: Stage set for MAS and map based gene cloning", in: *Abstracts 4th international rice genetics symposium, International Rice Research Institute, Philippines*), 22-27.

Khush, G.S., Bacalangco, E., and Ogawa, T. (1990). 18. A new gene for resistance to bacterial blight from O. longistaminata. *Rice Genet. News Lett* 7**,** 121-122.

Kim, S.-M. (2018). Identification of novel recessive gene xa44 (t) conferring resistance to bacterial blight races in rice by QTL linkage analysis using an SNP chip. *Theoretical and applied genetics* 131**,** 2733-2743.

Kim, S.-M., and Reinke, R.F. (2019). A novel resistance gene for bacterial blight in rice, Xa43 (t) identified by GWAS, confirmed by QTL mapping using a bi-parental population. *PloS one* 14(2)**,** e0211775.

Kim, S.-M., Suh, J.-P., Qin, Y., Noh, T.-H., Reinke, R.F., and Jena, K.K. (2015). Identification and fine-mapping of a new resistance gene, Xa40, conferring resistance to bacterial blight races in rice (Oryza sativa L.). *Theoretical and applied genetics* 128**,** 1933-1943.

Kottapalli, K.R., Lakshmi Narasu, M., and Jena, K.K. (2010). Effective strategy for pyramiding three bacterial blight resistance genes into fine grain rice cultivar, Samba Mahsuri, using sequence tagged site markers. *Biotechnology letters* 32**,** 989-996.

Kumar, A., Bernier, J., Verulkar, S., Lafitte, H., and Atlin, G. (2008). Breeding for drought tolerance: direct selection for yield, response to selection and use of drought-tolerant donors in upland and lowland-adapted populations. *Field Crops Research* 107(3)**,** 221-231.

Kumar, A., Jain, A., Sahu, R., Shrivastava, M., Nair, S., and Mohan, M. (2005). Genetic analysis of resistance genes for the rice gall midge in two rice genotypes. *Crop science* 45(4)**,** 1631-1635.

Kumar, P.N., Sujatha, K., Laha, G., Rao, K.S., Mishra, B., Viraktamath, B., et al. (2012). Identification and fine-mapping of Xa33, a novel gene for resistance to Xanthomonas oryzae pv. oryzae. *Phytopathology* 102(2)**,** 222-228.

Lanceras, J.C., Pantuwan, G., Jongdee, B., and Toojinda, T. (2004). Quantitative trait loci associated with drought tolerance at reproductive stage in rice. *Plant physiology* 135(1)**,** 384-399.

Lee, K., Rasabandith, S., Angeles, E., and Khush, G. (2003). Inheritance of resistance to bacterial blight in 21 cultivars of rice. *Phytopathology* 93(2)**,** 147-152.

Lee, S., Ahn, J., Cha, Y., Yun, D., Lee, M., Ko, J., et al. (2007). Mapping QTLs related to salinity tolerance of rice at the young seedling stage. *Plant Breeding* 126(1)**,** 43-46.

Lee, S., Costanzo, S., Jia, Y., Olsen, K.M., and Caicedo, A.L. (2009). Evolutionary dynamics of the genomic region around the blast resistance gene Pi-ta in AA genome Oryza species. *Genetics* 183(4)**,** 1315-1325.

Lima, J.M., Dass, A., Sahu, S., Behera, L., and Chauhan, D.K. (2007). A RAPD marker identified a susceptible specific locus for gall midge resistance gene in rice cultivar ARC5984. *Crop Protection* 26(9)**,** 1431-1435.

Lin, F., Chen, S., Que, Z., Wang, L., Liu, X., and Pan, Q. (2007). The blast resistance gene Pi37 encodes a nucleotide binding site–leucine-rich repeat protein and is a member of a resistance gene cluster on rice chromosome 1. *Genetics* 177(3)**,** 1871-1880.

Lin, X., Zhang, D., Xie, Y., Gao, H., and Zhang, Q. (1996). Identifying and mapping a new gene for bacterial blight resistance in rice based on RFLP markers. *Phytopathology* 86(11)**,** 1156-1159.

Liu, G., Mei, H., Yu, X., Zou, G., Liu, H., Hu, S., et al. (2008). QTL analysis of panicle neck diameter, a trait highly correlated with panicle size, under well-watered and drought conditions in rice (Oryza sativa L.). *Plant Science* 174(1)**,** 71-77.

Liu, X., Lin, F., Wang, L., and Pan, Q. (2007). The in silico map-based cloning of Pi36, a rice coiled-coil–nucleotide-binding site–leucine-rich repeat gene that confers race-specific resistance to the blast fungus. *Genetics* 176(4)**,** 2541-2549.

Luo, W., Huang, M., Guo, T., Xiao, W., Wang, J., Yang, G., et al. (2017). Marker‐assisted selection for rice blast resistance genes Pi2 and Pi9 through high‐resolution melting of a gene‐targeted amplicon. *Plant Breeding* 136(1)**,** 67-73.

Luo, Y., Ma, T., Zhang, A., Ong, K.H., Li, Z., Yang, J., et al. (2016). Marker-assisted breeding of the rice restorer line Wanhui 6725 for disease resistance, submergence tolerance and aromatic fragrance. *Rice* 9(1)**,** 1-13.

Ma, J., Lei, C., Xu, X., Hao, K., Wang, J., Cheng, Z., et al. (2015). Pi64, encoding a novel CC-NBS-LRR protein, confers resistance to leaf and neck blast in rice. *Molecular Plant-Microbe Interactions* 28(5)**,** 558-568.

Miao, L., Wang, C., Zheng, C., Che, J., Gao, Y., Wen, Y., et al. (2010). Molecular mapping of a new gene for resistance to rice bacterial blight. *Scientia Agricultura Sinica* 43(15)**,** 3051-3058.

Mishra, K.K., Vikram, P., Yadaw, R.B., Swamy, B.M., Dixit, S., Cruz, M.T.S., et al. (2013). qDTY 12. 1: a locus with a consistent effect on grain yield under drought in rice. *BMC genetics* 14**,** 1-10.

Mohan, M., Nair, S., Bentur, J., Rao, U.P., and Bennett, J. (1994). RFLP and RAPD mapping of the rice Gm2 gene that confers resistance to biotype 1 of gall midge (Orseolia oryzae). *Theoretical and Applied Genetics* 87**,** 782-788.

Mohapatra, S., Barik, S.R., Dash, P.K., Lenka, D., Pradhan, K.C., Raj K. R, R., et al. (2023). Molecular Breeding for Incorporation of Submergence Tolerance and Durable Bacterial Blight Resistance into the Popular Rice Variety ‘Ranidhan’. *Biomolecules* 13(2)**,** 198.

Mohapatra, S., Panda, A.K., Bastia, A.K., Mukherjee, A.K., Sanghamitra, P., Meher, J., et al. (2021). Development of submergence-tolerant, bacterial blight-resistant, and high-yielding near isogenic lines of popular variety,‘Swarna’through marker-assisted breeding approach. *Frontiers in Plant Science* 12**,** 672618.

Mohd Ikmal, A., Noraziyah, A.A.S., and Wickneswari, R. (2021). Incorporating drought and submergence tolerance QTL in Rice (Oryza sativa L.)—the effects under reproductive stage drought and vegetative stage submergence stresses. *Plants* 10(2)**,** 225.

Muthu, V., Abbai, R., Nallathambi, J., Rahman, H., Ramasamy, S., Kambale, R., et al. (2020). Pyramiding QTLs controlling tolerance against drought, salinity, and submergence in rice through marker assisted breeding. *PloS one* 15(1)**,** e0227421.

Nakai, H., Nakamura, K., Kuwahara, S., and Saito, M. (1988). Genetic studies of an induced rice mutant resistant to multiple races of bacterial leaf blight. *Rice Genetics Newsletter* 5**,** 101-103.

Nanda, A., Mohanty, S.K., Sovan Panda, R., Behera, L., Prakash, A., and Sahu, S.C. (2010). Flanking microsatellite markers for breeding varieties against Asian rice gall midge. *Tropical Plant Biology* 3**,** 219-226.

Neelam, K., Mahajan, R., Gupta, V., Bhatia, D., Gill, B.K., Komal, R., et al. (2020). High-resolution genetic mapping of a novel bacterial blight resistance gene xa-45 (t) identified from Oryza glaberrima and transferred to Oryza sativa. *Theoretical and Applied Genetics* 133**,** 689-705.

Ogawa, T., and Khush, G. (1989). Major genes for resistance to bacterial blight in rice. *Bacterial Blight of Rice. International Rice Research Institute, Manila, Philippines***,** 177-192.

Ogawa, T., Morinaka, T., Fujii, K., and Kimura, T. (1978). Inheritance of resistance of rice varieties Kogyoku and Java 14 to bacterial group V of Xanthomonas oryzae. *Japanese Journal of Phytopathology* 44(2)**,** 137-141.

Ogawa, T., and Yamamoto, T. (1986). "Inheritance of resistance to bacterial blight in rice," in *Rice Genetics I: (In 2 Parts)*. World Scientific), 471-479.

Okuyama, Y., Kanzaki, H., Abe, A., Yoshida, K., Tamiru, M., Saitoh, H., et al. (2011). A multifaceted genomics approach allows the isolation of the rice Pia‐blast resistance gene consisting of two adjacent NBS‐LRR protein genes. *The Plant Journal* 66(3)**,** 467-479.

Oladosu, Y., Rafii, M.Y., Arolu, F., Chukwu, S.C., Muhammad, I., Kareem, I., et al. (2020). Submergence tolerance in rice: Review of mechanism, breeding and, future prospects. *Sustainability* 12(4)**,** 1632.

Palanog, A.D., Swamy, B.M., Shamsudin, N.A.A., Dixit, S., Hernandez, J.E., Boromeo, T.H., et al. (2014). Grain yield QTLs with consistent-effect under reproductive-stage drought stress in rice. *Field Crops Research* 161**,** 46-54.

Petpisit, V., Khush, G.S., and Kauffman, H. (1977). Inheritance of resistance to bacterial blight in rice 1. *Crop science* 17(4)**,** 551-554.

Pidgeon, J.D., Ober, E.S., Qi, A., Clark, C.J., Royal, A., and Jaggard, K.W. (2006). Using multi-environment sugar beet variety trials to screen for drought tolerance. *Field crops research* 95(2-3)**,** 268-279.

Qu, S., Liu, G., Zhou, B., Bellizzi, M., Zeng, L., Dai, L., et al. (2006). The broad-spectrum blast resistance gene Pi9 encodes a nucleotide-binding site–leucine-rich repeat protein and is a member of a multigene family in rice. *Genetics* 172(3)**,** 1901-1914.

Ram, T., Laha, G., Gautam, S., Deen, R., Madhav, M.S., Brar, D., et al. (2010). Identification of new gene introgressed from Oryza brachyantha with broad-spectrum resistance to bacterial blight of rice in India. *Rice Genetics Newsletter* 25**,** 57.

Ramalingam, J., Palanisamy, S., Alagarasan, G., Renganathan, V.G., Ramanathan, A., and Saraswathi, R. (2020). Improvement of stable restorer lines for blast resistance through functional marker in rice (Oryza sativa L.). *Genes* 11(11)**,** 1266.

Reinke, R., Kim, S.-M., and Kim, B.-K. (2018). Developing japonica rice introgression lines with multiple resistance genes for brown planthopper, bacterial blight, rice blast, and rice stripe virus using molecular breeding. *Molecular Genetics and Genomics* 293**,** 1565-1575.

Rekha, G., Abhilash Kumar, V., Viraktamath, B., Pranathi, K., Kousik, M., Laxmi Prasanna, B., et al. (2018). Improvement of blast resistance of the popular high-yielding, medium slender-grain type, bacterial blight resistant rice variety, Improved Samba Mahsuri by marker-assisted breeding. *Journal of Plant Biochemistry and Biotechnology* 27**,** 463-472.

Ruengphayak, S., Chaichumpoo, E., Phromphan, S., Kamolsukyunyong, W., Sukhaket, W., Phuvanartnarubal, E., et al. (2015). Pseudo-backcrossing design for rapidly pyramiding multiple traits into a preferential rice variety. *Rice* 8(1)**,** 1-16.

Sabouri, H., Rezai, A., Moumeni, A., Kavousi, A., Katouzi, M., and Sabouri, A. (2009). QTLs mapping of physiological traits related to salt tolerance in young rice seedlings. *Biologia Plantarum* 53**,** 657-662.

Sakaguchi, S. (1967). Linkage studies on the resistance to bacterial leaf blight, Xanthomonas oryzae (Uyeda et Ishiyama) DOWSON, in rice. *Bull. Natl. Inst. Agr. Sci. Ser.* 16**,** 1-18.

Sanchez, A., Ilag, L., Yang, D., Brar, D., Ausubel, F., Khush, G., et al. (1999). Genetic and physical mapping of xa13, a recessive bacterial blight resistance gene in rice. *Theoretical and applied genetics* 98**,** 1022-1028.

Sandhu, N., Dixit, S., Swamy, B., Raman, A., Kumar, S., Singh, S., et al. (2019). Marker assisted breeding to develop multiple stress tolerant varieties for flood and drought prone areas. *Rice* 12**,** 1-16.

Sandhu, N., Singh, A., Dixit, S., Sta Cruz, M.T., Maturan, P.C., Jain, R.K., et al. (2014). Identification and mapping of stable QTL with main and epistasis effect on rice grain yield under upland drought stress. *BMC genetics* 15**,** 1-15.

Sardesai, N., Kumar, A., Rajyashri, K., Nair, S., and Mohan, M. (2002). Identification and mapping of an AFLP marker linked to Gm7, a gall midge resistance gene and its conversion to a SCAR marker for its utility in marker aided selection in rice. *Theoretical and Applied Genetics* 105**,** 691-698.

Sasayama, D., Okishio, T., Hirano, T., Fukayama, H., Hatanaka, T., Akimoto, M., et al. (2018). Internodal elongation under submergence in the Amazonian wild rice species Oryza glumaepatula: the growth response is induced by hypoxia but not by ethylene. *Plant Growth Regulation* 85**,** 123-132.

Sellamuthu, R., Ranganathan, C., and Serraj, R. (2015). Mapping QTLs for reproductive‐stage drought resistance traits using an advanced backcross population in upland rice. *Crop Science* 55(4)**,** 1524-1536.

Shang, J., Tao, Y., Chen, X., Zou, Y., Lei, C., Wang, J., et al. (2009). Identification of a new rice blast resistance gene, Pid3, by genomewide comparison of paired nucleotide-binding site–leucine-rich repeat genes and their pseudogene alleles between the two sequenced rice genomes. *Genetics* 182(4)**,** 1303-1311.

Sharma, T., Rai, A., Gupta, S., and Singh, N. (2010). Broad-spectrum blast resistance gene Pi-kh cloned from rice line Tetep designated as Pi54. *Journal of Plant Biochemistry and Biotechnology* 19**,** 87-89.

Shrivastava, M., Kumar, A., Bhandarkar, S., Shukla, B., and Agrawal, K. (2003). A new gene for resistance in rice to Asian rice gall midge (Orseolia oryzae Wood Mason) biotype 1 population at Raipur, India. *Euphytica* 130**,** 143-145.

Sidhu, G., Khush, G.S., and Mew, T. (1978). Genetic analysis of bacterial blight resistance in seventy-four cultivars of rice, Oryza sativa L. *Theoretical and Applied Genetics* 53**,** 105-111.

Singh, A., Gopalakrishnan, S., Singh, V., Prabhu, K., Mohapatra, T., Singh, N., et al. (2011). Marker assisted selection: a paradigm shift in Basmati breeding. *Indian Journal of Genetics and Plant Breeding* 71(2)**,** 120.

Singh, K., Vikal, Y., Singh, S., Leung, H., Dhaliwal, H., and Khush, G. (2002). 40. Mapping of bacterial blight resistance gene xa8 using microsatellite markers. *Rice Genetics Newsletter* 19**,** 94-97.

Singh, R., Khush, G., and Mew, T. (1983). A New Gene for Resistance to Bacterial Blight in Rice 1. *Crop Science* 23(3)**,** 558-560.

Singh, U.M., Dixit, S., Alam, S., Yadav, S., Prasanth, V.V., Singh, A.K., et al. (2022). Marker‐assisted forward breeding to develop a drought‐, bacterial‐leaf‐blight‐, and blast‐resistant rice cultivar. *The plant genome* 15(1)**,** e20170.

Singh, V.K., Singh, B.D., Kumar, A., Maurya, S., Krishnan, S.G., Vinod, K.K., et al. (2018). Marker-assisted introgression of Saltol QTL enhances seedling stage salt tolerance in the rice variety “Pusa Basmati 1”. *International journal of genomics* 2018.

Su, J., Wang, W., Han, J., Chen, S., Wang, C., Zeng, L., et al. (2015). Functional divergence of duplicated genes results in a novel blast resistance gene Pi50 at the Pi2/9 locus. *Theoretical and Applied Genetics* 128**,** 2213-2225.

Swamy, B.M., and Kumar, A. (2013). Genomics-based precision breeding approaches to improve drought tolerance in rice. *Biotechnology advances* 31(8)**,** 1308-1318.

Takagi, H., Uemura, A., Yaegashi, H., Tamiru, M., Abe, A., Mitsuoka, C., et al. (2013). M ut M ap‐G ap: whole‐genome resequencing of mutant F 2 progeny bulk combined with de novo assembly of gap regions identifies the rice blast resistance gene Pii. *New Phytologist* 200(1)**,** 276-283.

Takahashi, A., Hayashi, N., Miyao, A., and Hirochika, H. (2010). Unique features of the rice blast resistance Pish locus revealed by large scale retrotransposon-tagging. *BMC plant biology* 10**,** 1-14.

Tan, G.-X., Ren, X., Weng, Q.-M., Shi, Z.-Y., Zhu, L.-L., and He, G.-C. (2004). Mapping of a new resistance gene to bacterial blight in rice line introgressed from Oryza officinalis. *Yi chuan xue bao= Acta genetica Sinica* 31(7)**,** 724-729.

Taura, S., Ogawa, T., Tabien, R.E., Khush, G.S., Yoshimura, A., and Omura, T. (1992). Resistance gene of rice cultivar, Taichung Native 1 to Philippine races of bacterial blight pathogens. *Japanese Journal of Breeding* 42(2)**,** 195-201.

Trijatmiko, K.R., Supriyanta, Prasetiyono, J., Thomson, M.J., Vera Cruz, C.M., Moeljopawiro, S., et al. (2014). Meta-analysis of quantitative trait loci for grain yield and component traits under reproductive-stage drought stress in an upland rice population. *Molecular Breeding* 34**,** 283-295.

Venuprasad, R., Bool, M., Quiatchon, L., Sta Cruz, M., Amante, M., and Atlin, G. (2012). A large-effect QTL for rice grain yield under upland drought stress on chromosome 1. *Molecular Breeding* 30**,** 535-547.

Venuprasad, R., Dalid, C., Del Valle, M., Zhao, D., Espiritu, M., Sta Cruz, M., et al. (2009). Identification and characterization of large-effect quantitative trait loci for grain yield under lowland drought stress in rice using bulk-segregant analysis. *Theoretical and Applied Genetics* 120**,** 177-190.

Vikram, P., Swamy, B.M., Dixit, S., Ahmed, H., Cruz, M.S., Singh, A.K., et al. (2012). Bulk segregant analysis:“An effective approach for mapping consistent-effect drought grain yield QTLs in rice”. *Field Crops Research* 134**,** 185-192.

Vikram, P., Swamy, B.M., Dixit, S., Ahmed, H.U., Teresa Sta Cruz, M., Singh, A.K., et al. (2011). qDTY 1.1, a major QTL for rice grain yield under reproductive-stage drought stress with a consistent effect in multiple elite genetic backgrounds. *BMC genetics* 12**,** 1-15.

Wang, C., Wen, G., Lin, X., Liu, X., and Zhang, D. (2009). Identification and fine mapping of the new bacterial blight resistance gene, Xa31 (t), in rice. *European journal of plant pathology* 123**,** 235-240.

Wang, Y., Zang, J., Sun, Y., Ali, J., Xu, J., and Li, Z. (2013). Background‐independent quantitative trait loci for drought tolerance identified using advanced backcross introgression lines in rice. *Crop Science* 53(2)**,** 430-441.

Wang, Z.X., Yano, M., Yamanouchi, U., Iwamoto, M., Monna, L., Hayasaka, H., et al. (1999). The Pib gene for rice blast resistance belongs to the nucleotide binding and leucine‐rich repeat class of plant disease resistance genes. *The Plant Journal* 19(1)**,** 55-64.

Xu, X., Hayashi, N., Wang, C.-T., Fukuoka, S., Kawasaki, S., Takatsuji, H., et al. (2014). Rice blast resistance gene Pikahei-1 (t), a member of a resistance gene cluster on chromosome 4, encodes a nucleotide-binding site and leucine-rich repeat protein. *Molecular breeding* 34**,** 691-700.

Xuwei, J., Chunlian, W., and Qing, Y. (2007). Breeding of near-isogenic line CBB30 and molecular mapping of Xa30 (t), a new resistance gene to bacterial blight in rice. *Scientia Agricultura Sinica* 40(6).

Yang, T., Zhang, S., Zhao, J., Liu, Q., Huang, Z., Mao, X., et al. (2016). Identification and pyramiding of QTLs for cold tolerance at the bud bursting and the seedling stages by use of single segment substitution lines in rice (Oryza sativa L.). *Molecular Breeding* 36**,** 1-10.

Yoshimura, A., Mew, T., Khush, G., and Omura, T. (1983). Inheritance of resistance to bacterial blight in rice cultivar Cas 209. *Genetics* 73(10)**,** 1409-1412.

Yoshimura, S., Yamanouchi, U., Katayose, Y., Toki, S., Wang, Z.-X., Kono, I., et al. (1998). Expression of Xa1, a bacterial blight-resistance gene in rice, is induced by bacterial inoculation. *Proceedings of the National Academy of Sciences* 95(4)**,** 1663-1668.

Yoshimura, S., Yoshimura, A., Iwata, N., McCouch, S.R., Abenes, M.L., Baraoidan, M.R., et al. (1995). Tagging and combining bacterial blight resistance genes in rice using RAPD and RFLP markers. *Molecular breeding* 1**,** 375-387.

Yoshimura, S., Yoshimura, A., Saito, A., Kishimoto, N., Kawase, M., Yano, M., et al. (1992). RFLP analysis of introgressed chromosomal segments in three near-isogenic lines of rice for bacterial blight resistance genes, Xa-1, Xa-3 and Xa-4. *The Japanese Journal of Genetics* 67(1)**,** 29-37.

Yuan, B., Zhai, C., Wang, W., Zeng, X., Xu, X., Hu, H., et al. (2011). The Pik-p resistance to Magnaporthe oryzae in rice is mediated by a pair of closely linked CC-NBS-LRR genes. *Theoretical and applied genetics* 122**,** 1017-1028.

Zhai, C., Lin, F., Dong, Z., He, X., Yuan, B., Zeng, X., et al. (2011). The isolation and characterization of Pik, a rice blast resistance gene which emerged after rice domestication. *New Phytologist* 189(1)**,** 321-334.

Zhang, F., Zhuo, D.L., Zhang, F., Huang, L.Y., Wang, W.S., Xu, J.L., et al. (2015). Xa39, a novel dominant gene conferring broad‐spectrum resistance to Xanthomonas oryzae pv. oryzae in rice. *Plant Pathology* 64(3)**,** 568-575.

Zhang, G.-q., Angeles, E., Abenes, M., Khush, G., and Huang, N. (1996). RAPD and RFLP mapping of the bacterial blight resistance gene xa-13 in rice. *Theoretical and Applied Genetics* 93**,** 65-70.

Zhang, Q., Lin, S., Zhao, B., Wang, C., Yang, W., Zhou, Y., et al. (1998). Identification and tagging a new gene for resistance to bacterial blight (Xanthomonas oryzae pv. oryzae) from O. rufipogon. *Rice Genet Newsl* 15**,** 138-142.

Zhao, H., Wang, X., Jia, Y., Minkenberg, B., Wheatley, M., Fan, J., et al. (2018). The rice blast resistance gene Ptr encodes an atypical protein required for broad-spectrum disease resistance. *Nature communications* 9(1)**,** 2039.

Zhao, X.-Q., Xu, J.-L., Zhao, M., Lafitte, R., Zhu, L.-H., Fu, B.-Y., et al. (2008). QTLs affecting morph-physiological traits related to drought tolerance detected in overlapping introgression lines of rice (Oryza sativa L.). *Plant Science* 174(6)**,** 618-625.

Zheng, C.-K., Chun-Lian, W., Yuan-Jie, Y., Liang, Y.-T., and Kai-Jun, Z. (2009). Identification and molecular mapping of Xa32 (t), a novel resistance gene for bacterial blight (Xanthomonas oryzae pv. oryzae) in rice. *Acta Agronomica Sinica* 35(7)**,** 1173-1180.

Zhou, B., Qu, S., Liu, G., Dolan, M., Sakai, H., Lu, G., et al. (2006). The eight amino-acid differences within three leucine-rich repeats between Pi2 and Piz-t resistance proteins determine the resistance specificity to Magnaporthe grisea. *Molecular plant-microbe interactions* 19(11)**,** 1216-1228.
